# The Hypervariable Amino-Terminus of P1 Protease Modulates Potyviral Replication and Host Defense Responses

**DOI:** 10.1371/journal.ppat.1003985

**Published:** 2014-03-06

**Authors:** Fabio Pasin, Carmen Simón-Mateo, Juan Antonio García

**Affiliations:** Departamento de Genética Molecular de Plantas, Centro Nacional de Biotecnología (CNB-CSIC), Madrid, Spain; Agriculture and Agri-Food Canada, Canada

## Abstract

The replication of many RNA viruses involves the translation of polyproteins, whose processing by endopeptidases is a critical step for the release of functional subunits. P1 is the first protease encoded in plant potyvirus genomes; once activated by an as-yet-unknown host factor, it acts in *cis* on its own C-terminal end, hydrolyzing the P1-HCPro junction. Earlier research suggests that P1 cooperates with HCPro to inhibit host RNA silencing defenses. Using *Plum pox virus* as a model, we show that although P1 does not have a major direct role in RNA silencing suppression, it can indeed modulate HCPro function by its self-cleavage activity. To study P1 protease regulation, we used bioinformatic analysis and *in vitro* activity experiments to map the core C-terminal catalytic domain. We present evidence that the hypervariable region that precedes the protease domain is predicted as intrinsically disordered, and that it behaves as a negative regulator of P1 proteolytic activity in *in vitro* cleavage assays. In viral infections, removal of the P1 protease antagonistic regulator is associated with greater symptom severity, induction of salicylate-dependent pathogenesis-related proteins, and reduced viral loads. We suggest that fine modulation of a viral protease activity has evolved to keep viral amplification below host-detrimental levels, and thus to maintain higher long-term replicative capacity.

## Author Summary

RNA viruses are ideal systems for the study of population dynamics, relationships among pathogen traits such as fitness and virulence, and of host immune responses to pathogen attacks. Based on experimental evolution studies, early models equated parasite virulence with fitness. Some reports showed that viral virulence and fitness can be unlinked. Here we present evidence that the highly disordered N-terminal region of a potyviral P1 protein negatively regulates its self-cleavage activity. Removal of this regulator domain greatly affects viral infection, which is characterized by accelerated early replication and enhanced symptom severity. These properties are nonetheless associated with low viral accumulation and high induction of antiviral resistance markers. Finally, we propose that host-dependent regulation of P1 processing efficiency modulates viral virulence and alleviates the host antiviral responses.

## Introduction

Viruses are obligate parasite pathogens that hijack host factors to assure their own survival and propagation. Due to the limited coding capacity of their genome, viruses undertake distinct translational strategies [1]; one of most widely employed by RNA viruses involves polyprotein synthesis [2]. A full-length polyprotein is theoretically derived from a single translational event, compromising the timely expression of the individual viral cistrons. To overcome this possible drawback and successfully regulate replication, assembly and spreading stages, various post-translational mechanisms have evolved to modulate the spatial-temporal availability of functional viral subunits. For instance, it is not uncommon that the same polyprotein is hydrolyzed by several endopeptidases; cleavage kinetics are thus linked to enzyme processivity and, in *trans*-acting proteases, to the different affinity with the specific cleavage sites [3], [4]. Activation of viral proteases might depend on the availability of defined cell- or pathogen-encoded cofactors [5]–[7] and structural rearrangements that modulate substrate accessibility, as shown for the hepatitis C virus NS3 protease domain [8]. Zymogen activation and allostery are key regulatory mechanisms of trypsin-like proteases [9], a group of enzymes that is widespread in positive strand RNA viruses [10] and that includes P1 proteins of potyviruses [11].

Members of the genus *Potyvirus* (family *Potyviridae*) belong to the picorna-like supergroup and represent one of the largest groups of plant-infecting RNA viruses [12], [13]. Their single-stranded RNA genome is ≈10 kb in size and encodes a large polyprotein comprising (from N- to C-terminus) P1, HCPro, P3, 6K1, CI, 6K2, VPg, NIa-Pro, Nlb, and the coat protein (CP). An additional protein, P3N-PIPO, is originated by a frameshift in the P3 cistron [14], [15]. The cysteine protease NIa-Pro processes the C-terminal part of the polyprotein by seven cleavage events, while P1 and HCPro are responsible for their own release by a *cis* cleavage at their respective C-termini [16]–[18]. Once released, the mature P1 and HCPro C-terminal extremities are thought to be trapped in the active cleft, leading to autoinhibition of *trans* cleavage activity [18], [19]. Located at the beginning of the polyprotein, P1 was the last potyviral endopeptidase identified; inactivating mutations of its catalytic domain preclude virus viability [20], making P1 an attractive target for the development of antiviral tools. In contrast to the two other genome-encoded proteases, P1 relies on a still unidentified host factor for its activation [18]. Computational analysis of P1 potyviral proteins showed its great variability both in length and in amino acid sequence, and its diversification in potyviral species was thus associated with host specialization [21]. Although P1 involvement in the definition of virus host range was highlighted [22], [23], its specific contribution to potyviral infection is still unclear. Many functions were attributed to P1, such as cell-to-cell movement, systemic spread, and viral genome replication enhancement [reviewed in 24]; P1 was later shown to strengthen the RNA silencing suppressor activity of HCPro [25]–[28].

Here we used *Plum pox virus* (PPV) as a model system to study potyviral P1. Our results indicate that P1 and its protease domain have a positive role in potyvirus infection, independent of silencing suppression. Moreover, the *in vitro* and *in planta* data presented here suggest that host-dependent regulation of P1 self-cleavage activity has evolved to modulate the efficiency of viral infection and escape plant defense responses.

## Results

### P1 deficiency in PPV infection is not complemented by *Arabidopsis* plants with defective RNA silencing pathways

**Figure 1:**
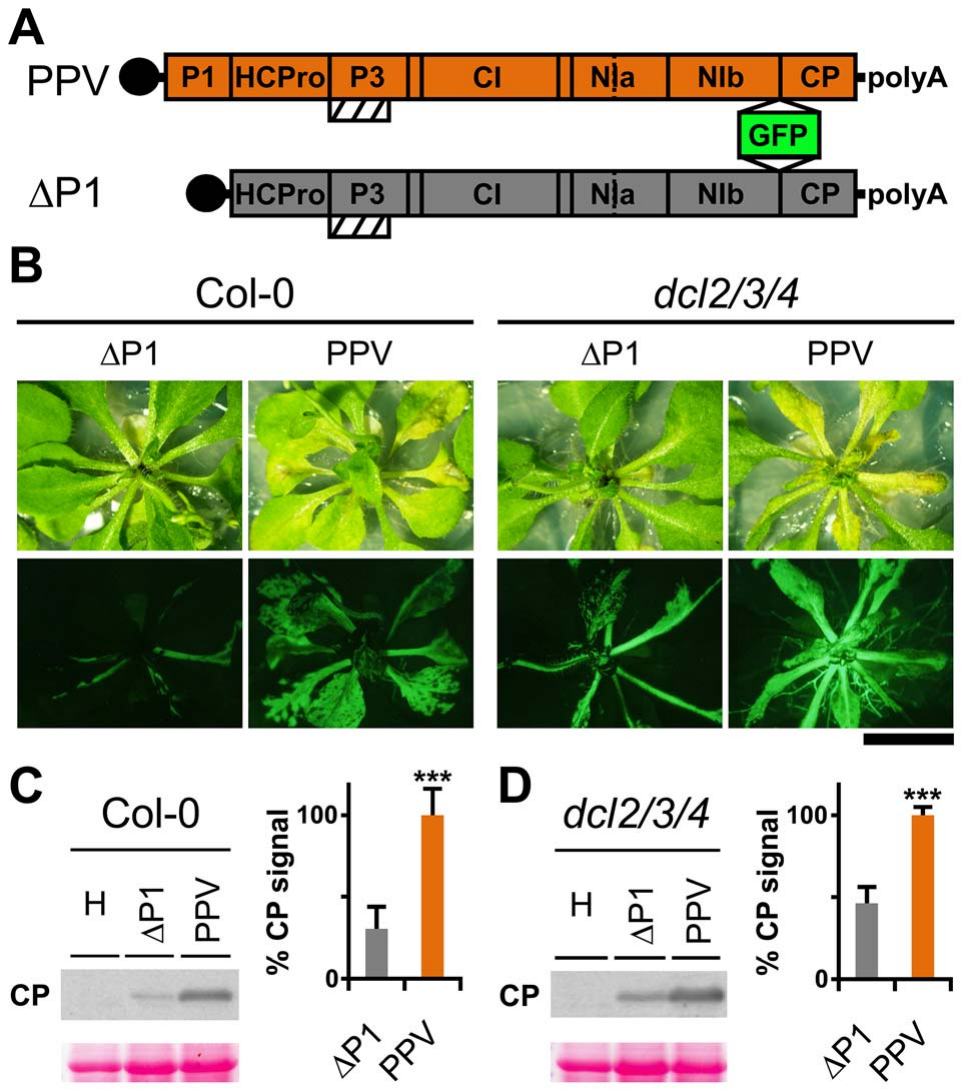
*Arabidopsis* mutant plants with defective RNA silencing pathways fail to rescue PPV ΔP1 amplification defects. (**A**) Representation of wild-type PPV and ΔP1, a PPV clone with deletion of the entire P1 sequence. The reporter sGFP(S65T) [103] gene is present between NIb and CP coding sequences of both viral clones. Boxes with diagonal lines indicate P3N-PIPO protein. (**B**) Symptoms and GFP fluorescence of *Arabidopsis* Col-0 and *dcl2/3/4* plants agro-inoculated with wild-type PPV or with ΔP1. Pictures were taken in an epifluorescence microscope at 15 dpi. Scale bar, 1 cm. (**C**) ΔP1 and wild-type PPV viral accumulation in *A. thaliana* Col-0 systemic leaves was assessed by anti-PPV CP (CP) western blot assay (15 dpi). Each lane represents a pool of samples used for signal quantification. H, healthy Col-0 plant sample. Relative CP signal intensities were plotted using average PPV value equal to 100. (**D**) ΔP1 and wild-type PPV viral accumulation in *A. thaliana dcl2/3/4* systemic leaves was assessed by anti-PPV CP (CP) western blot assay of upper non-inoculated leaves (15 dpi). Each lane represents a pool of samples used for signal quantification. H, healthy *dcl2/3/4* plant sample. Relative CP signal intensities were plotted using average PPV value equal to 100. Histograms show mean ±SD (*n* = 6 samples/condition, from two independent *Agrobacterium* cultures); *** *p*<0.001, Student's *t*-test. Ponceau red-stained blots are shown as loading control.

Genetic rescue of a *Turnip mosaic potyvirus* (TuMV) defective in RNA silencing suppression activity was reported in the *Arabidopsis thaliana* triple mutant line *dcl2-1 dcl3-1 dcl4-2* (*dcl2/3/4*) [29]. A PPV isolate adapted to *Nicotiana clevelandii* and able to infect herbaceous hosts [30], as well as its infectious cDNA clone encoding a green fluorescent protein (GFP) reporter gene, were described [31]. To study the P1 contribution in silencing suppression, we generated a viral clone that lacks the entire P1 sequence (ΔP1, Figure 1A), which we used to challenge *Arabidopsis* mutant plants with defective antiviral silencing pathways. At 15 days post-agro-inoculation (dpi), ΔP1-infected Col-0 and *dcl2/3/4* plants were almost symptomless, whereas PPV-infected Col-0 plants showed leaf chlorosis, which was stronger in the *dcl2/3/4* mutant line (Figure 1B). The presence of ΔP1 and wild-type PPV in systemic leaves was evidenced by GFP fluorescence detection (Figure 1B). Anti-PPV CP western blot analysis of systemically infected leaves showed that, in both host genotypes, viral accumulation was significantly higher in wild-type PPV than in ΔP1-infected plants (Figure 1C and D). The results confirmed that lack of P1 sequence did not compromise virus ability to replicate or to move systemically, as also shown for another potyvirus, *Tobacco etch virus* (TEV) [32]. Although ΔP1 appears to replicate better in the silencing deficient plants than in wild-type Col-0, the *dcl2/3/4* line did not restore the ΔP1 phenotype and viral accumulation to wild-type PPV levels (Figure 1B and D). Based on these results, the main role of P1 in viral amplification appears unrelated to RNA silencing suppression.

### The N-terminal region of potyviral P1 is predicted to be intrinsically disordered

To further analyze possible P1 functions, we coupled multiple sequence alignment and structural predictors for bioinformatic analysis of its sequence. Intrinsic protein disorder was estimated using DISOPRED2 [33] and MetaDisorderMD2 [34].

**Figure 2:**
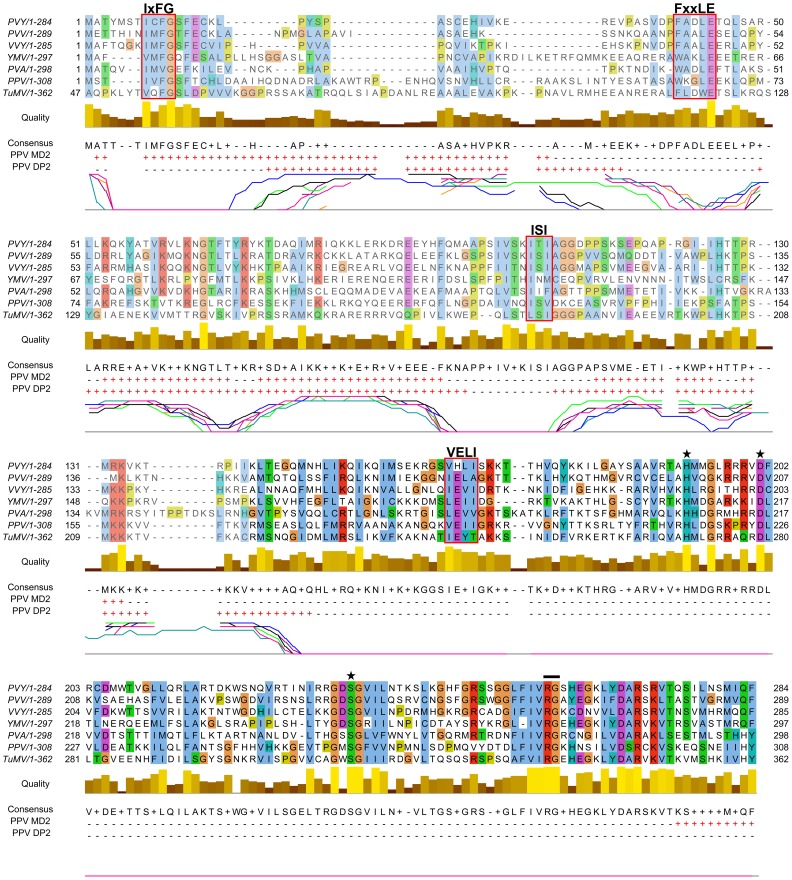
Alignment of P1 amino acid sequences from PPV and six reference potyviruses. GenBank accession numbers are reported in Table S1, in Text S1; amino acid background is assigned according to the ClustalX color scheme [120]. Residues aligning to PPV P1 minimal protease domain (aa 165-308, based on our analysis) are shown in black letters and bright-colored background. Alignment quality, based on BLOSUM62 scores, is shown as a bar graph (below). FxxLE and the previously reported conserved motifs IxFG, ISI, VELI are boxed. The protease conserved RG dipeptide is marked with black bar and the catalytic triad His, Asp and Ser is marked with stars. PPV MD2 and PPV DP2 lines show protein disorder prediction of PPV P1 sequence according to MetadisorderMD2 and DISOPRED2 analyses, respectively, where “+” are disordered and “−” ordered residues. Additionally, DISOPRED2 prediction confidence for each P1 sequence is plotted: PPV, black line; PVY, orange; PVA, turquoise; PVV, magenta; TuMV, blue; VVY, purple; YMV, green. The first 46 residues of TuMV P1 are hidden, as they do not align to the other accessions.

From the *in silico* analysis of P1 proteins from different potyviral species groups (Table S1, in Text S1), we identified FxxLE as a conserved motif in the P1 N-terminal region (Figure 2), in conjunction with the reported IxFG and ISI motifs [21]. The relatively well-conserved C-terminal regions of the proteins, which correspond to the protease domain (defined below) and include the VELI motif, are predicted mainly as structured. The residues from N-terminal sequence patches with least conservation are generally predicted to be intrinsically unstructured (Figure 2), suggesting that final protein conformation is the main evolutionary target, rather than maintenance of the primary sequence, as proposed [35]. The disorder predictions were further supported by P1 sequence analysis of *Scallion mosaic virus* and the monocotyledon-infecting *Sugarcane mosaic virus* group (Figure S1), which comprise the smallest known P1 sequences and are suggested to be at the base of the potyviral evolutionary tree [13].

### Involvement of P1 conserved motifs in protease self-cleavage

**Figure 3:**
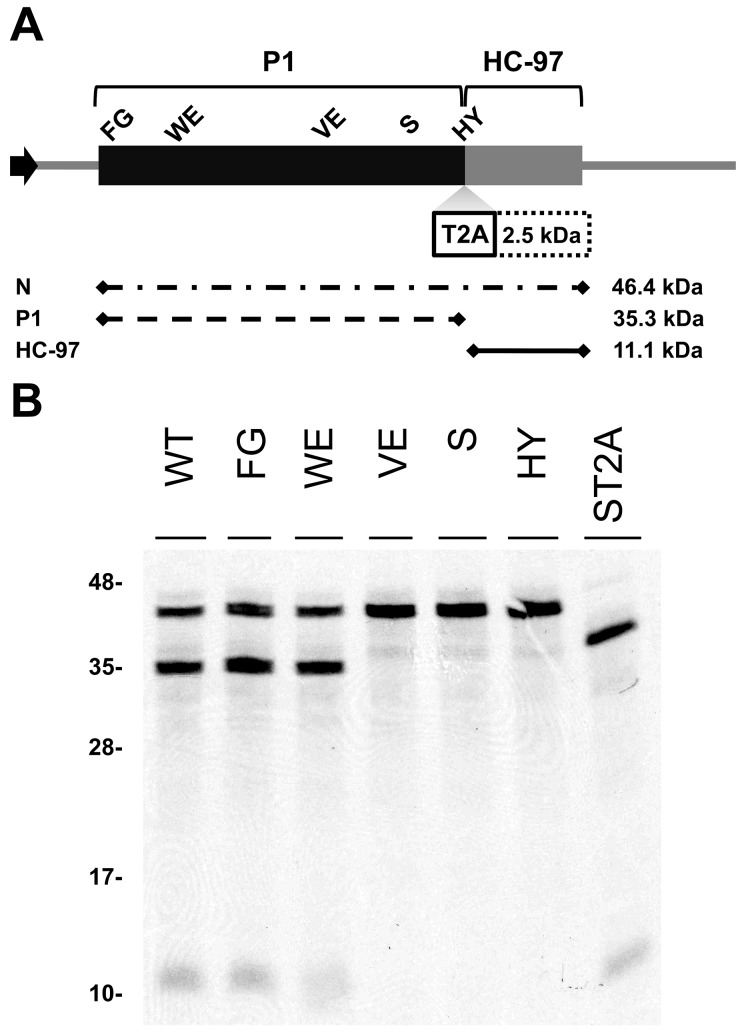
Involvement of P1 conserved motifs in protease cleavage. (**A**) Diagram of the DNA constructs used in the transcription reactions. T7 RNA polymerase promoter, black arrow, drives the cDNA of PPV 1-1361 genome nucleotides, comprising the 5′UTR and the ORF that codes for P1 (black box) and 97 N-terminal amino acids from HCPro (HC-97, grey box). HC-97 is followed by a stop codon and PPV 3′UTR cDNA. P1 residues chosen for alanine scanning are labeled (details below). When indicated, *Thosea asigna virus* 2A ‘self-cleaving’ peptide (T2A) was inserted in between P1 and HC-97. Below, expected translation products and their molecular weights are shown. (**B**) P1 self-cleavage was evaluated by *in vitro* translation in the wheat germ extract system. Labeled translation products were resolved by tricine-SDS-PAGE and the ^35^S signal detected. WT, wild-type P1; FG, FG-6,7-AA substitution of the IxFG motif; WE, W63A E67A substitution of the FxxLE motif; VE, VE-189,190-AA substitution of the VELI motif; S, replacement by alanine of the catalytic S259; HY, HY-307,308-AA substitution of P_2_ and P_1_ cleavage site residues; ST2A, catalytic S259A+T2A. Molecular weight markers are indicated (left).

To study the relevance of P1 motifs in protease activity, we mutated selected PPV P1 conserved amino acids to alanine (Figure 3A). The viral cDNA constructs were transcribed and *in vitro*-translated using the wheat germ extract (WGE) system. Replacement of catalytic S259 (S) and P_2_ and P_1_ cleavage site residues HY-307,308 (HY) with alanine impaired P1 protease self-cleavage, precluding release of the mature 35.3 kDa P1 protein and the 11.1 kDa HCPro fragment (HC-97) (Figure 3B). No proteolytic processing was detected in the construct bearing the VE-189,190-AA (VE) substitution in the VELI motif, indicating that it indeed belongs to the minimal protease domain, consistent with the disorder predictions. We found no appreciable differences between the wild-type P1 (WT) construct and those with FG-6,7-AA (FG) substitution in the IxFG motif or W63A E67A (WE) substitution in the FxxLE motif (Figure 3B), in accordance with the finding that the N-terminal region is dispensable for potyvirus P1 processing [18]. We tested whether the *Thosea asigna virus* 2A (T2A) “self-cleaving” peptide [36] could overcome P1 protease defects and restore P1-HCPro separation. We confirmed that T2A insertion between P1 S259A and HCPro (ST2A) restored precursor processing, which, considering the barely detectable level of the predicted 48.9 kDa uncleaved product, was more efficient than with the wild-type P1 construct.

### Involvement of P1 conserved motifs in viral infectivity

**Figure 4:**
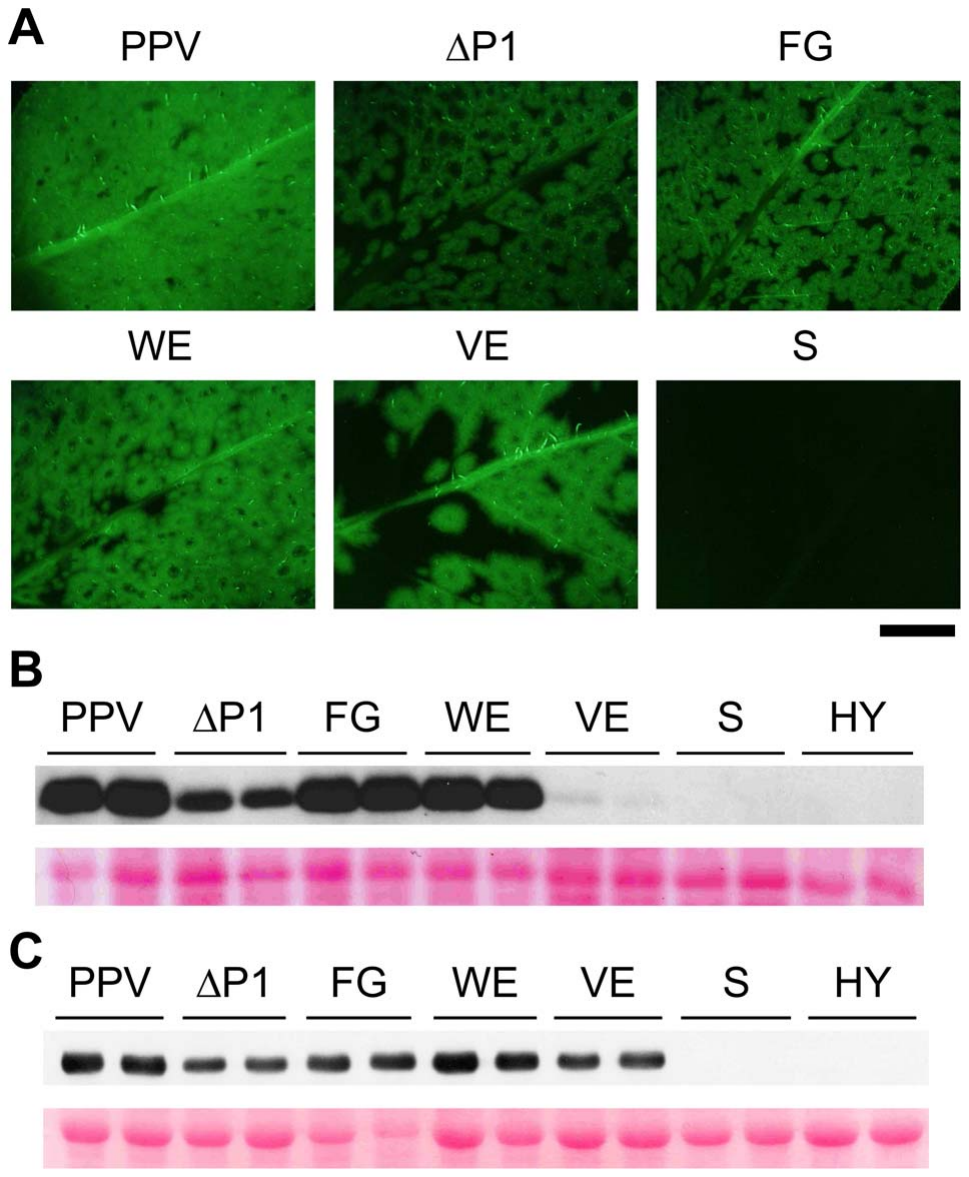
GFP fluorescence and viral accumulation of PPV cDNA clones with altered P1 sequence. *N. clevelandii* plants were agro-inoculated with the indicated binary vectors: PPV, pSN-PPV; ΔP1, pSN-PPV ΔP1; FG, pSN-PPV P1-FG; WE, pSN-PPV P1-WE; VE, pSN-PPV P1-VE; S, pSN-PPV P1-S; HY, pSN-PPV P1-HY. (**A**) Pictures of upper non-inoculated leaves were taken in an epifluorescence microscope (22 dpi). Scale bar, 1 cm. (**B**) Anti-PPV CP (CP) western blot assay of agro-inoculated *N. clevelandii* leaves (10 dpi); each lane corresponds to a single plant sample. (**C**) Anti-PPV CP (CP) western blot assay of upper non-inoculated *N. clevelandii* leaves (22 dpi); each lane corresponds to a single plant sample. Ponceau red-stained blots are shown as loading control.

To test their effect on viral infectivity, the P1 alanine substitutions used in *in vitro* translation were introduced into binary vectors bearing the full-length PPV cDNA. *N. clevelandii* plants were agro-infiltrated with *Agrobacterium* strains harboring pSN-PPV, pSN-PPV ΔP1, or pSN-PPV plasmids with point mutations in P1, and GFP fluorescence was monitored to follow the infection process of the derived viral clones. Infectious clones with full deletion (ΔP1) or point mutations in the P1 sequence (FG and WE) had characteristic ring-shaped GFP foci with no detectable fluorescence in the center compared to the wild-type virus (PPV) (Figure 4A). A similar phenotype was observed in a *Potato virus A* (PVA) clone with the GFP gene inserted into the P1 region [37], suggesting that in this recombinant potyvirus, P1 function was also affected. Plants challenged with clones harboring the S or the HY mutations, which had no proteolytic activity in the *in vitro* translation assays, did not show GFP fluorescence, infection symptoms or CP accumulation (Figure 4A–C and not shown).

Although P1 VE-189,190-AA replacement led to no detectable cleavage in WGE experiments (Figure 3), a faint CP accumulation signal (compared to wild-type PPV) was visible in leaves agro-infiltrated with the VE clone harboring the same mutations, and the CP signal was greatly enhanced in systemically infected leaves (Figure 4B and C). These leaves showed the characteristic ring-shaped GFP foci typical of the other infectious P1 mutants (Figure 4A). The results led us to hypothesize that partial reversion might occur, restoring P1 protease activity. Western blot performed using anti-PPV HCPro antibody confirmed correct P1-HCPro processing in the upper non-inoculated leaves of plants agro-inoculated with the VE mutant clone (Figure S2A). Samples of systemically infected leaves from these plants were further subjected to RT-PCR amplification of a PPV genome fragment spanning the mutations introduced. Though the E190A mutation was maintained, we detected reversion of V189A to the wild-type residue at 22 dpi (Figure S2B). It is likely that, in contrast to clones S and HY, clone VE maintained minimal P1 self-processing activity sufficient to initiate viral replication and to select viral mutant progeny with improved cleavage efficiency. This is consistent with a report of a TEV clone in which disruption of the (at that time undescribed) VELI motif appeared to preclude P1 self-processing *in vitro*. A virus harboring the same mutation was infectious, however, and able to move systemically, with a marked delay compared to the parental virus [20].

At the same time point at which P1 VE-189,190-AA reversion was detected (22 dpi), the rest of the mutations introduced were stably maintained in both FG and WE infectious clones (verified by RT-PCR and sequencing, not shown), confirming that mutations of N-terminal motifs are less detrimental than protease defects.

### P1 protease defects impair the silencing suppressor activity of HCPro

**Figure 5:**
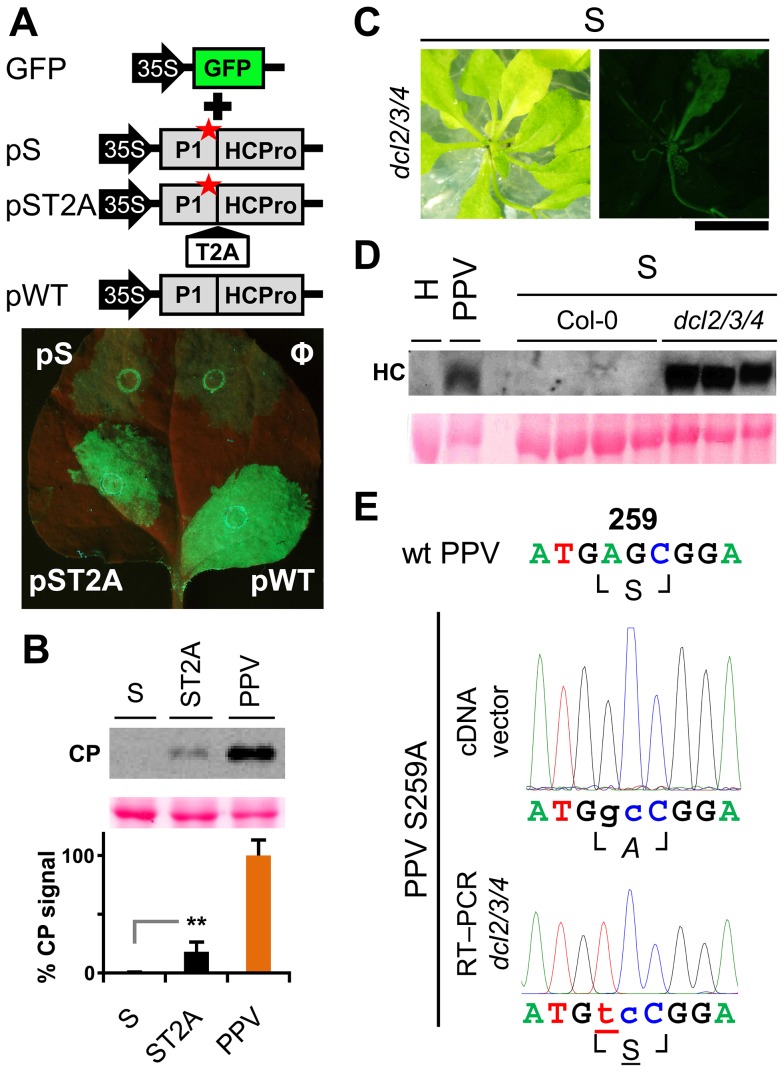
RNA silencing suppression is impaired by the lack of separation between P1 and HCPro. (**A**) The transient RNA silencing assay was done by co-infiltrating *N. benthamiana* leaf tissue with an *Agrobacterium* p35S:GFP culture and cultures of *Agrobacterium* without plasmid (Ф) or containing pSN.5 P1-S (pS, producing P1 S259A+HCPro), pSN.5 P1-ST2A (pST2A, producing P1 S259A+T2A+HCPro), or pSN.5 PPV (pWT, producing wild-type P1+HCPro). Picture was taken under UV light (6 dpa). Scale bar, 1 cm. (**B**) *N. clevelandii* plants were agro-inoculated with wild-type PPV and viral clones S (P1 S259 replaced by alanine) and ST2A (P1 S259A+T2A peptide). Viral accumulation in inoculated leaves was assessed by anti-PPV CP (CP) immunoblot analysis (6 dpi); each lane represents a pool of samples used for signal quantification. Relative CP signal intensities were plotted using average PPV value equal to 100. Histograms show mean ± SD (*n* = 4 samples/condition, from two independent *Agrobacterium* cultures); ** *p*<0.01, Student's *t*-test. Ponceau red-stained blot is shown as loading control. (**C**) Symptoms and GFP fluorescence of *Arabidopsis dcl2/3/4* plant agro-inoculated with PPV mutant clone S (P1 S259 replaced by alanine). Pictures were taken in an epifluorescence microscope at 23 dpi. Scale bar, 1 cm. (**D**) Anti-PPV HCPro (HC) western blot assay of agro-inoculated *Arabidopsis* plants. Samples of upper non-inoculated leaves were collected (23 dpi). H, healthy *dcl2/3/4* plant; PPV, pool of 16 Col-0 plants challenged with wild-type PPV; S, plants challenged with viral clone S, where Col-0 samples are pools of four plants and *dcl2/3/4* are pools of two/three *dcl2/3/4* plants showing GFP fluorescence. Ponceau red-stained blot is shown as loading control. (**E**) RT-PCR of *A. thaliana dcl2/3/4* plants challenged with viral clone S (23 dpi). A PPV cDNA fragment encompassing the mutated regions (nt 1–1197 of the PPV genome) was amplified by RT-PCR from a pool of three plants and analyzed by DNA sequencing. Sanger sequencing results of the RT-PCR fragment and of the inoculated plasmid DNA are shown. The wild-type PPV sequence is shown as reference. Nucleotides that differ from the wild-type sequence are indicated in lower case. The nucleotide that leads to reversion of the amino acid codon is underlined. Encoded amino acids are indicated beneath each nucleotide sequence and between box-drawing characters. The mutated residue is in italic (alanine), reverted residue underlined.

Viability of the TEV clones altered by mutations in the P1 protease can be restored by insertion of a surrogate cleavage site recognized by the TEV NIa protease [20], or in transgenic plants expressing the P1-HCPro cistron [32]. To test whether these rescue strategies complement defects in HCPro function rather than in P1, we performed a transient RNA silencing assay in *N. benthamiana*. Leaves were co-infiltrated with an *Agrobacterium* strain bearing p35S:GFP as a silencing reporter and *Agrobacterium* strains containing the PPV silencing suppressor HCPro preceded by the wild-type P1 (pWT), by P1 with an alanine replacement of the catalytic S259 (pS) or by P1 S259A plus T2A (pST2A). GFP fluorescence was visible in all agroinfiltrated patches at 3 days post-agro-infiltration (dpa) (not shown). At 6 dpa, there were no differences between the empty control and the S construct. In contrast, bright fluorescence as a result of silencing suppression was maintained when HCPro was effectively released by P1 protease activity or by the ribosome skipping mechanism of T2A (Figure 5A).

PPV viral clone ST2A, into which we inserted the T2A peptide sequence between P1 S259A and HCPro, was able to initiate viral replication (6 dpi; Figure 5B) and move systemically despite the P1 protease-inactivating mutation (Figure S2C; maintenance of P1 S259A substitution was verified by RT-PCR and sequencing, not shown). The presence of T2A was nevertheless insufficient to fully complement viral defects, since the ST2A GFP fluorescence phenotype and CP accumulation levels differed considerably from wild-type PPV (Figure 5B, Figure S2C and D).

To further validate these results, *A. thaliana* Col-0 and the RNA silencing-defective *dcl2/3/4* line were agro-inoculated with the PPV S259A mutant (S), using wild-type PPV as control. As predicted, wild-type PPV infected 16 of 16 challenged plants of both the mutant line and its wild-type background, at 23 dpi. Although we could not detect replication of the S mutant in Col-0 by GFP fluorescence or western blot analysis, GFP fluorescence was observed in 11 of 16 *dcl2/3/4* plants (Figure 5C). In spite of the cleavage-disturbing mutation in the S cDNA clone, P1-HCPro proteolytic separation was rescued in *dcl2/3/4* plants inoculated with PPV S259A, as verified by western blot assay of HCPro (Figure 5D). RT-PCR analysis of samples collected at 23 dpi confirmed that the original serine and the protease activity were restored in the viral progeny, since the wild-type serine 259 codon AGC, which was mutated to alanine GCC in the PPV S259A cDNA clone, was further mutated to serine UCC (Figure 5E). The fact that viral reversion mutations are promptly selected in PPV S259A-infected plants suggests that P1, in addition to the release of an active silencing suppressor, has further function(s).

### The first 164 N-terminal residues are dispensable for PPV P1 self-processing and act as a negative regulator in *in vitro* cleavage assay

**Figure 6:**
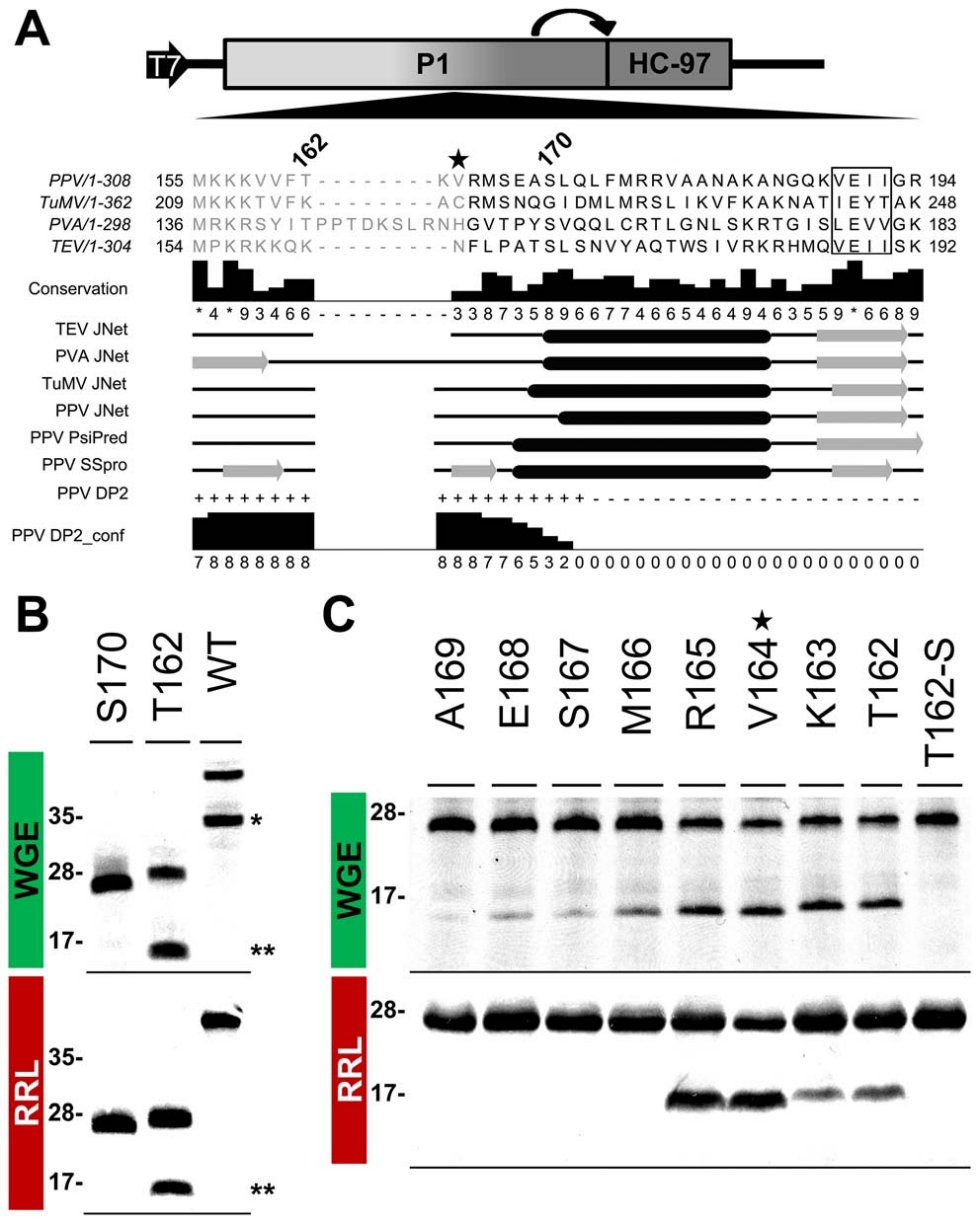
Mapping of P1 minimal protease domain and assessment of cleavage activity in eukaryotic translation systems. (**A**) Diagram of the DNA constructs used in the transcription reactions, as detailed in Figure 3, and partial alignment of P1 from PPV and three reference potyviruses (see GenBank accession numbers in Table S1, in Text S1). Beside each virus name, P1 size is indicated. Positions of the first and last P1 amino acid shown in the alignment are indicated. Numbers above the alignment indicate the position of PPV P1 residues selected for a preliminary trial. The star marks V164, the first amino acid maintained in the mutant clone used for the viral replication assay. The VELI motif is boxed. Residues aligning to the PPV P1 minimal protease domain (see below) are shown in black letters, the remaining residues in gray letters. Alignment conservation bars are displayed. JNet, PsiPred and SSPro secondary structure predictions are presented, α-helices, black ovals; β-sheets, grey arrows. PPV DP2, disorder prediction of PPV P1 sequence according to DISOPRED2, where “+” are disordered and “−” ordered residues. PPV DP2_conf, DISOPRED2 prediction confidence. (**B**) P1 self-cleavage was evaluated by *in vitro* translation. N-terminal deletions were named according the first amino acid maintained (in addition to the initial methionine) in the truncated P1 protein tested. Labeled translation products were resolved by tricine-SDS-PAGE and the ^35^S signal detected. Products of reactions performed using wheat germ extract (WGE; top panel) and rabbit reticulocyte lysate (RRL; bottom). Molecular weight markers are shown (left). Double asterisks indicate the 16.7 kDa P1 fragment released by the T162 construct. Single asterisk indicates the full-length P1 protein (35.3 kDa) released by the WT construct. (**C**) Fine mapping of the PPV P1 protease domain. Nomenclature as in (B). T162-S also presents alanine replacement of the catalytic S259 residue. WGE reaction, top panel; RRL, bottom. Star marks V164, as the corresponding P1 N-terminal deletion was introduced into a PPV cDNA clone for the viral replication assay. Molecular weight markers are indicated (left).

According to MEROPS classification [38], potyviral P1 serine protease belongs to subclan PA(S), whose representative member, trypsin, is synthesized as the inactive precursor trypsinogen, with a disordered loop partially obstructing the substrate-binding cleft [39]. Supported by the intrinsic disorder prediction of the P1 N-terminal region and since a non-viral factor is needed for P1 protease activation [18], we tested whether P1 also fits the trypsin model. Previous studies on TEV [40], sequence alignment, secondary structure predictions, intrinsic disorder confidence, as well as the finding that in PPV, the VE-189,190-AA substitution disturbed P1 self-processing, were considered in choosing PPV P1 residues T162 and S170 as truncation points for a preliminary trial (Figure 6A). N-terminal deletion constructs were made by removing the P1 sequence upstream of the codon for each amino acid selected, except for the initial methionine. To test P1 protease activity, we used *in vitro* translation in WGE and rabbit reticulocyte lysate (RRL) systems. The full-length P1 (WT) construct released the mature 35.3 kDa P1 protein in WGE but not in the RRL system (Figure 6B, single asterisk), as anticipated [18]. Nonetheless, the T162 deletion construct successfully self-cleaved in both WGE and RRL systems, as shown by release of the 16.7 kDa P1 processed fragment (Figure 6B, double asterisks). The S170 construct lacked activity in both *in vitro* translation systems, and only its uncleaved 27.0 kDa precursor was detectable (Figure 6B), suggesting that the P1 residue delimiting the N-terminal minimal protease domain is located between positions 162 and 169.

To fine-map this boundary, additional single amino acid deletions were engineered, transcribed, and tested by *in vitro* translation. As a further control, the catalytic S259A mutation was included in the T162 construct to rule out misleading non-specific protein degradation. Efficient cleavage activity was maintained in both WGE and RRL after deletion of P1 N-terminal amino acids 2-164 (Figure 6C, R165 construct), confirmed by release of the ≈16 kDa P1 mature fragment. Further truncations of the protease domain led to the drastic disappearance of protease activity in RRL, and gradually decreasing efficiency in WGE. The control protease catalytic mutant T162-S showed only a band corresponding to the unprocessed 27.8 kDa precursor. The results indicate that the protease catalytic domain is correctly folded even in RRL, and that the first 164 N-terminal residues are not only dispensable for this activity, but show an antagonistic effect on P1 self-processing in RRL.

### Removal of the P1 N-terminus enhances early amplification of PPV

**Figure 7:**
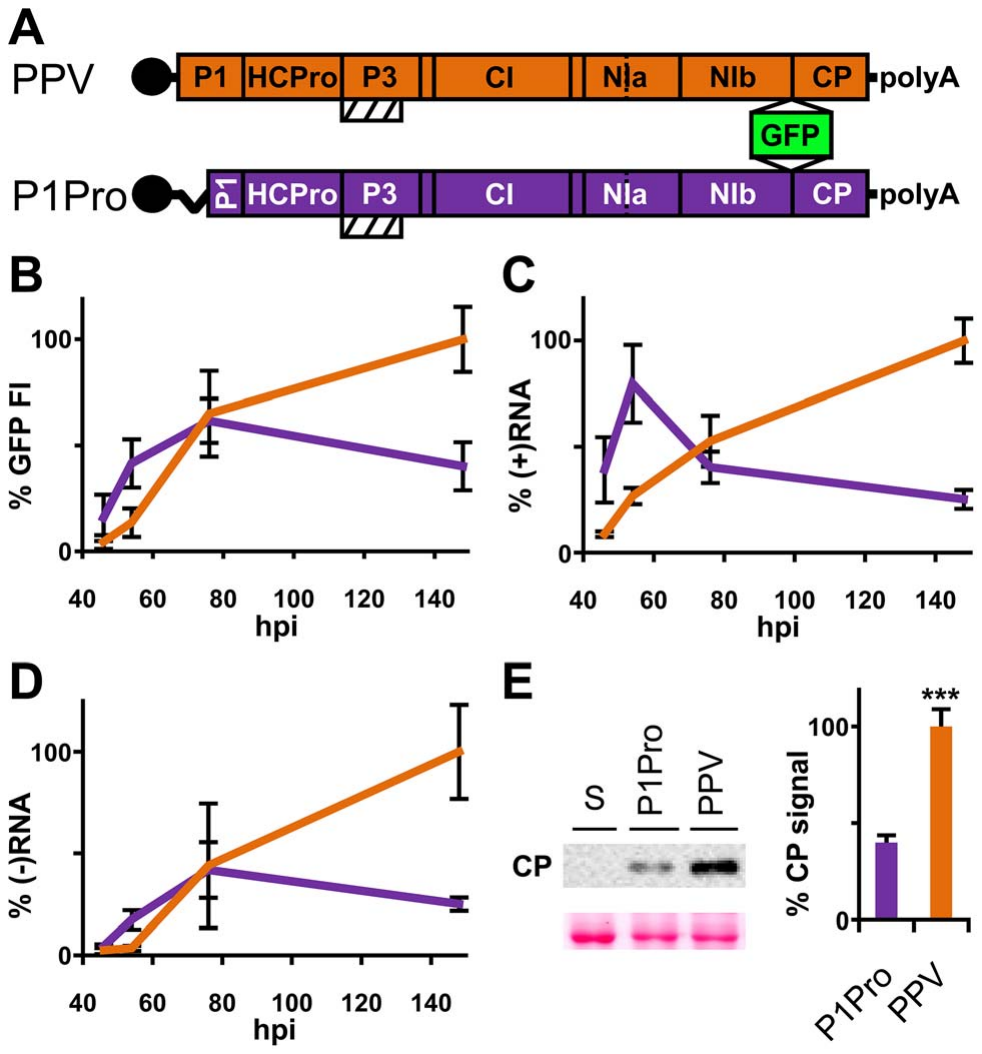
Early amplification dynamics for wild-type PPV and P1Pro viral clones. (**A**) Leaves of three-week-old *N. clevelandii* plants were agro-inoculated with pSN-PPV (wild-type PPV) or pSN-PPV P1Pro[V164] (P1Pro, in which P1 amino acids 2-163 were deleted). The reporter sGFP(S65T) [103] gene is present between NIb and CP coding sequences of both viral clones. Boxes with diagonal lines indicate P3N-PIPO protein. (**B**) GFP fluorescent intensity (FI) from inoculated leaves was quantified in a fluorometer and plotted using average wild-type PPV value at 148 hpi equal to 100. Line graph shows mean ± SD (*n* = 16 samples/condition, from two independent *Agrobacterium* cultures); colors as in (A). (**C**) Amount of viral (+)RNA from inoculated leaves was quantified by RT-qPCR and plotted using average wild-type PPV value at 148 hpi equal to 100. Line graph shows mean ± SD (*n* = 3/4 biological replicates, from two independent *Agrobacterium* cultures); colors as in (A). (**D**) Amount of viral (−)RNA from inoculated leaves was quantified by RT-qPCR and plotted using average wild-type PPV value at 148 hpi equal to 100. Line graph shows mean ± SD (*n* = 4 biological replicates, from two independent *Agrobacterium* cultures); colors as in (A). (**E**) Anti-PPV CP (CP) western blot assay of inoculated leaves (148 hpi); each lane represents a pool of samples used for signal quantification. S, sample of plants inoculated with the control viral clone pSN-PPV P1-S. Relative CP signal intensities were plotted using average PPV value equal to 100. Histograms show mean ± SD (*n* = 4 samples/condition, from two independent *Agrobacterium* cultures); *** *p*<0.001, Student's *t*-test. Ponceau red-stained blot is shown as loading control.

The PPV cDNA clone pSN-PPV P1Pro[V164], lacking P1 amino acids 2-163 (P1Pro, Figure 7A), was engineered to evaluate how a P1 protease free of its antagonistic N-terminal region affects viral replication. We deleted P1 residues upstream of position 164, since the *in vitro* cleavage assays showed that the V164 construct was the most efficient P1 truncation tested (Figure 6C, star). Various reporter genes cloned into viral infectious cDNAs were used to quantify potyviral genome replication rates [41], [42]. Taking advantage of the GFP marker inserted in our PPV clones, fluorescence intensity (FI) signals were used to monitor viral amplification kinetics. Compared to the wild-type PPV, FI levels were significantly higher in leaves agro-inoculated with P1Pro clone at 46 h post-agro-inoculation (hpi) and 54 hpi (3.6- and 3.1-fold, respectively). At later time points, while the FI of wild-type PPV continued to increase over the 6-day time course, that of P1Pro slowed at 3 dpi and dropped to 0.4 times the level of parental PPV at 148 hpi (Figure 7B). To support the GFP FI results, growth dynamics were analyzed in viral RNA by RT-qPCR (Figure 7C and D). Fluorescence quantification values correlated positively with the amounts of PPV (+)RNA and (−)RNA (Spearman's R_S_ = 0.857 and R_S_ = 0.976, respectively). Viral amount at the end of the growth curve was further assessed by anti-PPV CP western blot, and confirmed that significantly less P1Pro virus accumulated than wild-type PPV at 148 hpi (Figure 7E). These data demonstrate that initiation of PPV amplification is delayed transiently in the presence of the P1 N-terminal end, which is necessary to maintain higher viral accumulation rates in the long term.

To test whether the PPV P1Pro viral decline depends on defects in RNA silencing suppression, we performed a transient agro-infiltration assay in *N. benthamiana* and *N. clevelandii* plants. GFP was used as silencing reporter, and was stably maintained in leaf patches co-expressing HCPro preceded either by the wild-type P1 (pWT) or by the P1 lacking amino acids 2–163 (pP1Pro), as evaluated by GFP FI and western blot analysis (Figure S3). This suggests that deletion of the P1 N-terminal region does not impair HCPro silencing suppressor activity.

### Removal of the P1 N-terminus enhances PPV symptom severity and is associated with pathogenesis-related (PR) protein accumulation

**Figure 8:**
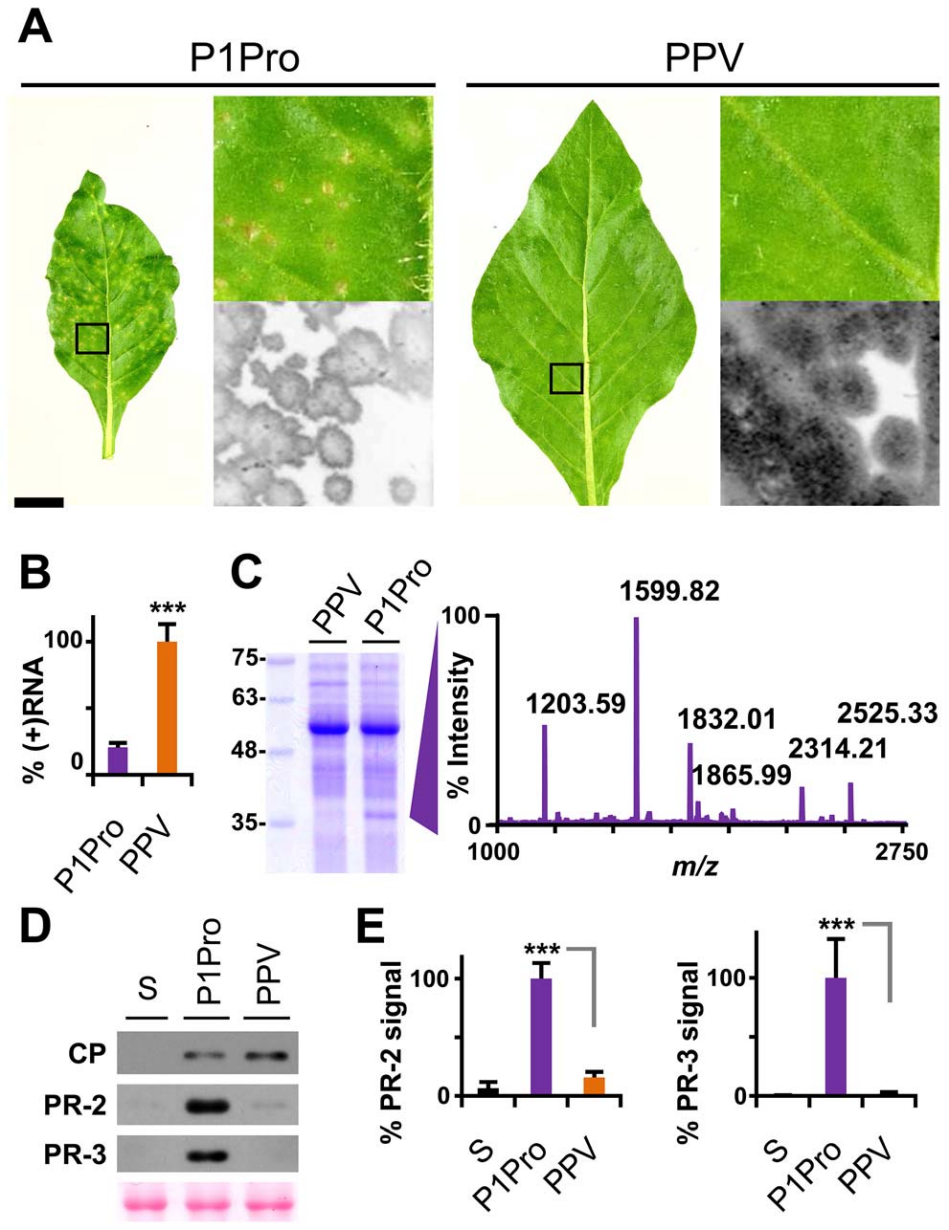
Symptoms and viral accumulation of PPV lacking P1 N-terminal residues. *N. clevelandii* plants were agro-inoculated with wild-type PPV or P1Pro, pSN-PPV P1Pro[V164] in which P1 amino acids 2–163 were deleted; data were collected at 21 dpi. (**A**) Pictures of *N. clevelandii* systemic leaves. Boxed leaf areas were magnified and their GFP fluorescence, acquired by laser scanning, is shown. Scale bar, 2.5 cm in whole leaf pictures, 0.4 cm in details. (**B**) Amount of viral (+)RNA from systemically infected leaves was quantified by RT-qPCR and plotted using average wild-type PPV value equal to 100. Histograms show mean ± SD (*n* = 4 biological replicates, from two independent *Agrobacterium* cultures); *** *p*<0.001, Student's *t*-test. (**C**) Total protein extracts from systemic leaf samples were resolved by glycine-SDS-PAGE and Coomassie blue stained, molecular weight markers are indicated (left). The ≈35–37 kDa P1Pro-specific band was analyzed by MALDI-TOF and its peptide mass fingerprint is shown. Intensity in the *y* axis; *m/z* values are indicated for the six P1Pro sample peptides further analyzed by MS/MS. (**D**) Anti-PPV CP (CP), anti-PR-2 (PR-2) and anti-PR-3 (PR-3) western blot assays of upper non-inoculated leaves; each lane represents a pool of samples used for signal quantification. S, sample of plants inoculated with the control viral clone pSN-PPV P1-S. Ponceau red-stained blot as loading control of protein extracts. (**E**) Relative PR-2 and PR-3 signal intensities were plotted using average P1Pro value equal to 100. Histograms show mean ± SD (*n* = 4 samples/condition, from two independent *Agrobacterium* cultures); *** *p*<0.001, Student's *t*-test.

To confirm and complement the early growth kinetics, we agro-inoculated *N. clevelandii* plants with PPV P1Pro and evaluated systemic infection. Symptoms in these plants, which were soon much more severe than those of plants inoculated with wild-type PPV, included marked stunting and necrotic lesions in the center of the larger chlorotic spots. As for the ΔP1 and P1 mutants (Figure 4), GFP fluorescence faded with progressing focus expansion in P1Pro-infected leaves, with no detectable signal in the center, and showing the characteristic ring shape before the appearance of necrosis (Figure 8A). RT-qPCR analysis confirmed that in systemically infected leaves (21 dpi), despite more severe symptoms, less viral RNA accumulated in P1Pro-infected plants than in wild-type PPV samples (Figure 8B). The identity of infecting viruses was confirmed by RT-PCR that encompassed the deletion, and by sequencing (not shown).

At the protein level, P1Pro-infected plant extracts were characterized by a specific band migrating at ≈35-37 kDa, which was unappreciable in wild-type PPV protein extracts and was thus analyzed by mass spectrometry (MS) (Figure 8C). In the MALDI-TOF retrieved spectrum, the P1Pro band showed six prominent peaks (Figure 8C), which were analyzed by MS/MS to obtain their peptide fragmentation fingerprint. Database searching allowed assignment of the *m/z* 1203.59 fragment to residues 326–335 of *N. tabacum* acidic PR-2 isoform GI9 (GenBank accession no. P23547.1). After removal of the signal peptide, this protein has a reported molecular weight of 34.8 kDa [43], in good agreement with the electrophoretic mobility of the P1Pro-specific band. Sequence of the other five major peaks was defined *de novo* (Figure S4A), since database identification of was unsuccessful due to unavailability of the host genome sequence (*N. clevelandii*). Based on MS/MS spectra, we verified that all five matched a glucan endo-1,3-β-D-glucosidase, EC 3.2.1.39, belonging to class II of the PR-2 family [44], [45], and that the minor amino acid changes identified were consistent with acidic PR-2 sequence variability in Solanaceae species (Figure S4B).

PR proteins of different families overaccumulate in tobacco plants that show hypersensitivity to *Tobacco mosaic virus* (TMV) [46]–[49], but also after bacterial and fungal infection and in response to abiotic stress [50]. We first confirmed the MS results by western blot analysis for PR-2; next, to test whether PR-2 induction is a specific P1-associated defense mechanism or part of a broader stress response, we assessed class II PR-3 protein expression in infected *N. clevelandii* plants (Figure 8D). We found that, despite the lower viral CP levels, PR-2 and PR-3 accumulation was significantly higher in P1Pro- compared to wild-type PPV-infected plants (Figure 8E).

### Immune response to PPV P1Pro is attenuated by downregulation of salicylic acid signaling

In tobacco plants, exogenous salicylic acid (SA) treatment induces class II PR-2 and class II PR-3 transcription [48], [51], [52], and activates the resistance responses associated to TMV-induced hypersensitivity [53], [54]. Downregulation of SA accumulation and systemic acquired resistance was reported in transgenic plants that express the bacterial salicylate-hydroxylase gene *nahG* [55].

**Figure 9:**
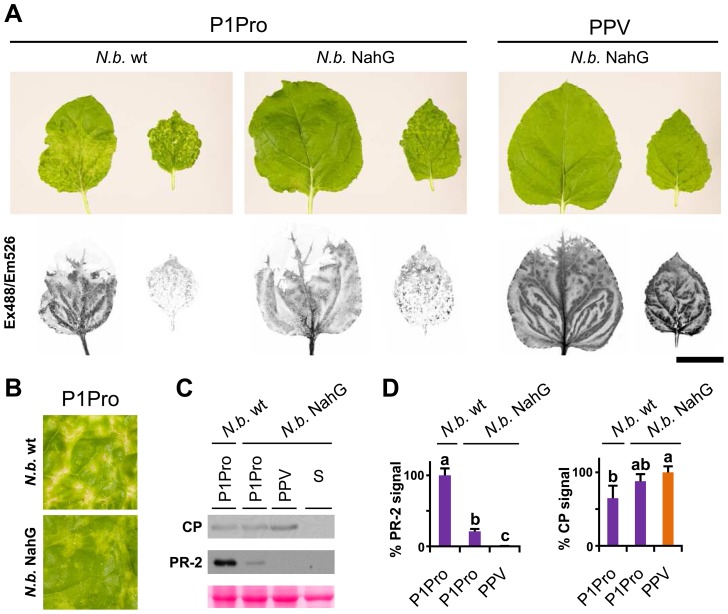
Viral accumulation and immune response induction in NahG-expressing *N. benthamiana*. (**A**) Wild-type (N.b. wt) and NahG-expressing (N.b. NahG) *N. benthamiana* plants were agro-inoculated with wild-type PPV and P1Pro viral clones. Pictures of systemic leaves were taken at 10 dpi. GFP fluorescence, acquired by laser scanning, is shown. Scale bar, 4 cm. (**B**) Details of (A) showing symptoms of wild-type and NahG-expressing plants infected with the P1Pro viral clone. (**C**) Anti-PPV CP (CP) and anti-PR-2 (PR-2) western blot analysis of upper non-inoculated leaves of wild-type and NahG-expressing plants (12 dpi); each lane represents a pool of samples used for signal quantification. S, sample of plants inoculated with the control viral clone pSN-PPV P1-S. Below, blot stained with Ponceau red as loading control of protein extracts. (**D**) Relative PR-2 and CP signal intensities were plotted using average P1Pro or wild-type PPV value equal to 100, respectively. Histograms show mean ± SD (*n* = 4 samples/condition, from two independent *Agrobacterium* cultures). Bars with different letters are statistically significant, *p*<0.05, one-way Anova and Tukey's HSD test.

In *N. benthamiana*, while wild-type PPV-infected plants appeared almost symptomless, PPV P1Pro-infected plants showed extended chlorosis and necrotic lesions, similar to those observed in *N. clevelandii* (Figure 9A). We therefore used transgenic *N. benthamiana* NahG plants [56] to evaluate the SA contribution in the PPV P1Pro host immune response. In accordance with previous studies [56], downregulation of SA signaling had no appreciable effect on the wild-type PPV phenotype. In contrast, in P1Pro-infected NahG plants, systemic symptom severity was attenuated (10 dpi; Figure 9A and B). This result was supported by a sharp reduction in PR-2 protein accumulation in P1Pro-infected NahG plants and a weak increase in viral load, estimated by anti-PPV CP western blot analysis (12 dpi; Figure 9C and D). These data suggest that although SA-mediated antiviral pathways have only a minor role in wild-type PPV infection, they take part in P1Pro immune responses.

### Several stress response proteins are upregulated in P1Pro-infected NahG plants

**Figure 10:**
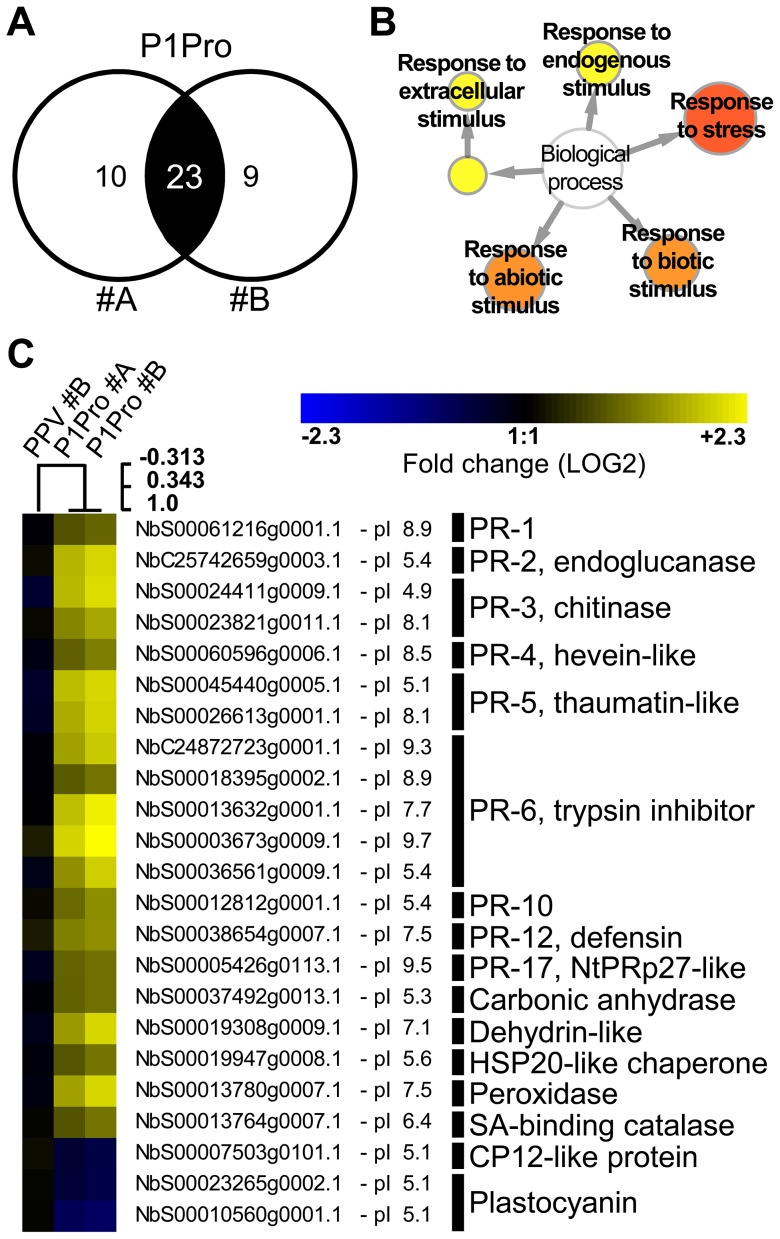
Quantitative proteomic analysis of PPV P1Pro-infected NahG plants. (**A**) Venn diagram of proteins whose abundance was significantly changed in P1Pro biological replicates #A and #B, relative to wild-type PPV sample #A. A false discovery rate of <5% was applied as statistical cut-off. Numbers in the non-overlapping areas represent unique significant proteins of each biological replica; the number in the overlapping area, 23, shows the common significant proteins for further analysis. (**B**) Map of gene ontology terms significantly enriched in 23 dysregulated proteins common to both P1Pro replicates. Relevant biological process nodes are colored from yellow to red, where red is the most significantly overrepresented category. In white, non-significant node. (**C**) Heat map of 23 significantly differentially regulated proteins common to P1Pro replicates #A and #B. Fold change was calculated using PPV #A values as 1, and expressed in LOG2. Quantification values of PPV biological replica #B are shown as control. Sample tree was built by hierarchical clustering analysis. Beside each *N. benthamiana* accession ID, protein isoelectric point is indicated. Brief protein descriptions are reported on the right; PR proteins are grouped by families according van Loon *et al.* [50].

In NahG-expressing plants (i) P1Pro-induced chlorosis was more accentuated than in wild-type PPV-infected plants (Figure 9A), (ii) although lower than the wild-type host, PR-2 abundance in P1Pro samples was significantly higher than in wild-type PPV, and (iii) PR-2 reduction was insufficient to fully restore P1Pro viral accumulation to parental PPV levels (Figure 9D). These findings prompted us to further investigate P1Pro-related defense responses after downregulation of SA signaling. To identify proteins whose abundance was significantly changed in P1Pro-infected NahG plants relative to wild-type PPV-infected NahG plants, we performed quantitative proteomic analysis using isobaric tag labeling (iTRAQ) and liquid chromatography (LC)-MS/MS [57]. A draft sequence of the *N. benthamiana* genome was released [58], [59], and a search against its predicted protein database enabled us to identify more than a thousand non-redundant proteins. Of these, 23 were considered to accumulate differentially in P1Pro versus wild-type PPV-infected plant samples, since they were found in both P1Pro biological replicates #A and #B, with a false discovery rate <5% as statistical cut-off (Figure 10A). Gene ontology term enrichment analysis showed that, according to plant symptoms, the 23 dysregulated proteins associated significantly with the GO term “response to stress” (GO ID: 6950, *p*<0.0001; Figure 10B). In Figure 10C, we present a heat map of these quantified P1Pro proteins with their average iTRAQ ratios (expressed in LOG2) relative to wild-type PPV biological replica #A. As predicted, P1Pro biological replicates #A and #B are grouped in the same hierarchical cluster, which differs from PPV biological replica #B. In P1Pro samples, several proteins from different PR families were more abundant than in wild-type PPV. These include a class II PR-2 (in accordance with the western blot result; Figure 9C) and other acidic members, which are regulated in tobacco mainly by SA [50], [52], and basic counterparts such as the basic PR-1, whose transcription is effectively activated by ethylene [60]–[64]. Abscisic acid and osmotic stress are reported to induce expression of basic PR-5 [50], [65], as well as of dehydrin-like proteins [66]. The contribution of oxidative stress in the SA-dependent response to P1Pro is underlined by the detection of a peroxidase and a catalase; the tobacco homologue of the latter was initially identified as a SA-binding protein [67]. Only three of the 23 differentially accumulated host proteins were downregulated in P1Pro; these include two plastocyanins and a CP12-like accession, proteins essential for photosynthesis [68], [69]. These data suggest that in the transgenic host, an important component of SA signaling is maintained and/or that its downregulation is partially compensated by alternative defense components.

## Discussion

Besides the self-cleavage activity intrinsic to its C-terminal end, other activities of potyviral P1 and its real contributions to viral replication remained vague, so much so that P1 earned the appellative “mysterious protein” in a recent review [24].

In concomitance with the discovery that HCPro inhibits posttranscriptional gene silencing, it was suggested that P1 could act in conjunction with and strengthen the silencing suppressor activity of HCPro [25], [26]. A characteristic ring-shaped GFP focus phenotype was related in tobamovirus infections with deficiency in RNA silencing suppression [70], [71]. The same GFP phenotype was distinctive of all our PPV infectious clones with P1 sequence deletions and point mutations. In contrast, our *Arabidopsis dcl2/3/4* complementation data demonstrate a major function for P1 independent of RNA silencing suppression. The ring GFP phenotype of P1 mutants might thus be related not only to a silencing suppression defect, but also to other altered functions. Recently, the silencing suppressor-enhancing effect of P1 was attributed to *cis* elements that improve translation efficiency, rather than to complementary activity of the P1 protein [72].

Our results nonetheless show that P1 is unquestionably involved in RNA silencing by limiting HCPro function. Deficiencies in polyprotein processing at the P1-HCPro junction abrogated PPV infectivity in wild-type plants, as also reported for TEV [20], and were partially complemented by insertion of the T2A “self-cleaving” peptide. To further define the nature of this viral weakness, we used a transient RNA silencing assay to show that for correct silencing suppression, P1 must be effectively separated from HCPro, whether mediated by its own protease domain or by the ribosome skipping mechanism of T2A. In plants with impaired antiviral RNA silencing machineries the PPV S259A clone, with a cleavage-inactive P1 protease, recovered infectivity. The selection of viral revertant progeny demonstrated that strong evolutionary pressure for proteolytic competence of P1 was maintained, despite the host mutant background. The lack of separation between P1 and HCPro probably impairs not only the RNA silencing suppressor activity, but also other important viral functions.

Of the mature proteins encoded by the potyviral genome, P1 presents the greatest variability in length and in amino acid sequence [73]. *In silico* analysis showed that the limited primary sequence conservation of the P1 N-terminal region is associated with residues predicted to be part of intrinsically disordered loops. The relevance of unfolded regions in the proteolytic maturation of viral polyproteins has been shown [6], [7], and flexible loops in peptidase precursors act in many cases as protease activation switches [39]. Previous results and those reported here show successful self-cleavage activity of potyviral P1 after *in vitro* translation only in the WGE system, but not in RRL. In consequence, it was suggested that a non-viral factor, present in WGE and absent in RRL, might be involved in correct folding of the protease domain or presentation of the cleavage site [18]. Our evaluation of the cleavage performance of P1 N-terminal end truncations demonstrates that the P1 minimal protease domain is active in WGE but also in RRL, suggesting that both eukaryotic translation systems support correct folding and activity of the P1 protease domain. Hence, the P1 N-terminal residues are not only dispensable for HCPro release, but also act as a negative regulator of P1 self-processing.

In the context of viral infection, removal of the P1 protease antagonistic extension accelerated early viral replication and was followed by enhancement of symptom severity, with the appearance of plant stunting and necrotic lesions that did not characterize wild-type PPV. The greater aggressiveness did not parallel viral loads, however, as viral RNA and CP levels in plants systemically infected with the deletion mutant were lower than in wild-type PPV-infected plants. Lack of positive correlation between symptom severity and fitness is reported for several viral systems, including potyviral infections [74]–[76]. This can be justified by the crossing of a virulence threshold, which would result in higher induction of immune responses or excessive host debilitation [77], [78]. Accordingly, the necrotic phenotype that characterizes PPV P1Pro was associated with overaccumulation of class II PR-2 and PR-3 proteins. Acidic PR-2 and PR-3 family members are indicators of SA-mediated responses to biotic stress and are hallmarks of systemic acquired resistance [48], [50], [79]. In a transgenic NahG-expressing plants, a decrease in SA signaling attenuated PPV P1Pro symptom severity. P1Pro-induced chlorosis was nonetheless more pronounced than in wild-type PPV-infected plants. Complex hormone crosstalk follows plant infection [80], and compounds other than SA operate in the defense against pathogens [81]–[83]. Quantitative proteomic analysis of NahG plants infected with the P1Pro viral clone allowed us to identify a large set of stress response-associated proteins whose abundance was significantly altered compared to wild-type PPV. This includes upregulation of members of different PR families whose transcription is reported to be activated by SA but also by ethylene, and further modulated by abscisic acid [50], as well as rearrangement of the antioxidant system, and downregulation of photosynthetic components, similar to other studies [84]–[86]. Coordinated action of both SA-dependent and -independent pathways probably contributes to the immune response activated by PPV P1Pro. In turn, the N-terminal extremity of the P1 protease, absent in P1Pro but maintained in wild-type PPV, helps to bypass induction of host defense responses. Several plant viruses interfere with salicylate pathways [87]–[89], and it would be of interest to determine whether P1 also has an active role in suppressing basal host defenses independent of its HCPro activity-modulating effect.

Considering that (i) defects in P1 self-cleavage preclude viral viability, (ii) viral RNA silencing suppression is impaired by the lack of separation between P1 and HCPro, (iii) P1 residues 1–164 are predicted to be mainly disordered, and negatively affect P1-HCPro processing in RRL, (iv) PPV early amplification dynamics are enhanced by removal of the P1 N-terminal end, (v) increased defense responses are associated with deletion of the P1 N-terminus, and (vi) removal of the P1 N-terminus reduces viral accumulation, we propose a model by which P1 acts to fine-tune potyviral replication by sensing specific host effectors. The presence of these cofactors leads to activation of the P1 protease domain and release of the silencing suppressor HCPro. The virus can thus effectively counteract host antiviral RNA silencing defenses and replicate successfully, but it can also stimulate additional defense responses. In an alternative scenario in which the cofactor is limited, P1-HCPro separation is restricted, RNA silencing suppression activity is disturbed, and some decrease in viral replication can therefore be predicted. We speculate that this mechanism holds viral replication below a level that would be detrimental to the host cell and reduces triggering of host immune responses, thus maintaining higher long-term replicative capacity.

An example of restriction in a virus infection step to avoid host defense responses is the regulation of autoprocessing of the NS2 protein of the pestivirus *Bovine viral diarrhea virus* (BVDV), which is dependent on the host chaperone Jiv [90]. Limiting amounts of Jiv cofactor result in accumulation of uncleaved NS2-3 and RNA replication arrest. Like the necrogenic interaction of PPV P1Pro, the disengagement of NS2 self-cleavage from cellular Jiv leads to a switch from a non-cytopathogenic to a cytopathogenic biotype, underlining the importance of the temporal modulation of NS2-3 processing [91], [92].

To understand the regulation of P1-HCPro processing, identification of the P1 activating host effector will be a priority for future studies. Moreover, P1 protease multifunctionality was postulated [20], [93] and indications that leader proteases retain activities independent of proteolytic processing can be found in several viruses [94]–[96]. Given the limited infection efficiency of the PPV ΔP1 viral mutant and the effects of mutagenesis of P1 conserved motifs, it is likely that P1 protein or its RNA coding sequence has additional unrevealed roles in potyviral replication.

## Materials and Methods

### Sequence analysis

Potyviral P1 sequences were visualized and analyzed using Jalview [97]. Multiple sequence alignments were made using MUSCLE [98]. Protein disorder predictions were made with DISOPRED2 [33], setting a false positive rate threshold of 10%, and MetaDisorderMD2 [34]. Secondary structure predictions used the JNet algorithm [99] included in the Jalview package, PsiPred [100] and SSpro [101].

### Plant materials

*Nicotiana clevelandii, N. benthamiana, N. benthamiana* NahG [56] (kindly provided by Prof. H.S. Guo, Chinese Academy of Sciences, Beijing, China, and Dr. F. Tenllado, CIB-CSIC, Madrid, Spain), *Arabidopsis thaliana* Col-0 and its triple mutant line *dcl2-1 dcl3-1 dcl4-2* [102] (kindly provided by Prof. J.C. Carrington, CGRB, Oregon State University, Corvallis, OR, USA) were used. *Nicotiana* plants were grown in a greenhouse maintained at a 16 h light/8 h dark photoperiod, temperature range 19–23°C. *Arabidopsis* plants were grown in the greenhouse or *in vitro* (see conditions below).

### DNA plasmids and constructs

A full-length cDNA copy of a PPV isolate [30], tagged with sGFP(S65T) [103] and inserted in the pBINPPV-NK-GFP binary plasmid, was reported [104]. Cloning in binary vectors is often constrained by the availability of unique restriction sites or, in the case of the Gateway technology [105], the maintenance of λ integrase recombination sites in the final constructs. To overcome these limitations, pBINPPV-NK-GFP was engineered to obtain the pSN-ccdB plasmid. Once linearized, pSN-ccdB is suitable for one-step seamless replacement of the PPV 5′UTR and the P1 cDNA sequence by Gibson assembly [106]; it was therefore used as a backbone for all the viral cDNA clones in this study. A detailed description of the viral vectors, together with *in vitro* translation and agro-infiltration constructs, can be found in Text S1 of the Supporting Information.

### *In vitro* transcription and translation

*In vitro* transcription reactions were performed with the T7-Scribe Standard RNA IVT kit (CELLSCRIPT), including final DNase I digestion. RNA was purified by organic extraction/ammonium acetate precipitation. Quality was assessed by NanoDrop (Thermo Fisher Scientific) and by gel electrophoresis, with final concentration adjusted to 1 µg/µL. *In vitro* translation was carried out in the presence of L-[^35^S]methionine and L-[^35^S]cysteine (PerkinElmer) using the WGE or the RRL translation system (Promega), according to manufacturer's instructions. Samples were resolved in 12% tricine-SDS-PAGE [107] and the signal detected by PhosphoImager or autoradiography.

### *Arabidopsis in vitro* agro-infection

*Arabidopsis* seeds were surface sterilized, kept at 4°C for three days, and sown *in vitro* on Murashige and Skoog medium [108] with MES and vitamins (Duchefa), adjusted to pH 5.7 with KOH and supplemented with 1% (w/v) sucrose and 0.7% (w/v) Bacto Agar (Difco). Six days after germination, plants were transferred to half-strength Murashige and Skoog medium with MES and vitamins, adjusted to pH 5.7 with KOH and supplemented with 1% (w/v) sucrose and 0.8% (w/v) Bacto Agar. At 9–10 days post-germination, agro-infection was performed as follows. *Agrobacterium* cultures were induced as described [28], the final OD_600_ was adjusted to 1.5, forceps were dipped into the culture, and the two youngest plantlet leaves were pierced. Forceps were flame-sterilized between cultures of different bacterial clones. Plates were sealed with gas-permeable tape (Millipore) and maintained in a growth chamber at 16 h light/8 h dark photoperiod, temperature 21±1°C, 60% relative humidity.

### *Nicotiana* plant agro-infiltration

The transient RNA silencing assay and agro-infiltration of *N. benthamiana* and *N. clevelandii* plants with *A. tumefaciens* strain C58C1 bearing the indicated plasmids, were as described [28]. The viral replication assay was conducted in three-week-old *N. clevelandii* plants following reported agroinfiltration and sampling guidelines [109], with the exception that a saturating concentration of *Agrobacterium* was used.

### GFP fluorescence imaging and quantification

GFP fluorescence was monitored under long-wavelength UV light (Black Ray, model B 100 AP) and photographed using a Nikon D1X digital camera with a 62E 022 filter. Alternatively, images were acquired under a MZ FLIII epifluorescence microscope (Leica) using a GFP3 filter (excitation and barrier filters at 470/40 nm and 525/50 nm, respectively) and photographed with an Olympus DP70 digital camera. When indicated, GFP fluorescence was acquired by laser scanning (Typhoon 9400, GE Healthcare; laser 488 nm, intensity 450 V; 526 nm short-pass emission filter).

GFP fluorescent intensity quantification was carried out placing individual 5.0 mm-diameter leaf discs in a black 96-well plate. Signals were acquired in an Appliskan (Thermo Fisher Scientific) or Victor X2 (PerkinElmer) plate readers with the following settings: measurement time 500 ms, excitation and emission wavelengths of 485/10 nm and 535/20 nm, respectively.

### Western blot assays

Plant tissue was ground in a mortar in liquid nitrogen or homogenized in a TissueLyzer bead mill (Qiagen). Total proteins were extracted in 150 mM Tris-HCl pH 7.5, 6 M urea, 2% (w/v) SDS, 5% (v/v) glycerol and 5% (v/v) β-mercaptoethanol; heat denatured (96°C, 5 min) and centrifuged (14000 rpm, 10 min) to remove cell debris. Protein samples were separated by glycine-SDS-PAGE and electroblotted onto a nitrocellulose membrane. Ponceau red staining was used to control protein loading equivalence. Proteins were detected using anti-PPV CP and anti-PPV HCPro rabbit sera, and anti-GFP monoclonal antibody (clones 7.1 and 13.1, Roche) as primary antibodies. Antibodies raised against tobacco class II PR-2 [44] and class II PR-3 proteins [47] were kindly provided by Dr. T. Heitz (IBMP-CNRS, Strasbourg, France). Horseradish peroxidase-conjugated goat anti-rabbit IgG (Jackson) or sheep anti-mouse IgG (GE Healthcare) were used as secondary antibody. Immunostained proteins were visualized by enhanced chemiluminescence detection with a LiteABlot kit (Euroclone). For signal quantification, chemioluminescence was acquired in a ChemiDoc XRS imager (BioRad) and analyzed with ImageJ software [110].

### RT-PCR and RT-qPCR

Total RNA was extracted with the FavorPrep Plant Total RNA Mini kit (Favorgen). Fragments spanning the PPV 5′UTR, and the coding sequences of P1 and the HCPro N-terminus were amplified with the Titan One Tube RT-PCR kit (Roche) using primers 1595_F/1597_R (Table S2, in Text S1). When indicated, fragments were purified using the FavorPrep Gel/PCR Purification kit (Favorgen) and DNA was sequenced.

Strand-specific quantification of PPV RNA was done for at least three biological replicates per condition using tagged cDNA primers in the RT step [111]–[113], and will be detailed elsewhere (in preparation). Briefly, equal amounts of DNAseI-treated total RNA were used for cDNA synthesis using Superscript III (Invitrogen) and primer Q26_R or Q29_F to transcribe cDNA from positive and negative PPV genomes, respectively. Technical triplicate qPCR reactions were prepared using HOT FIREPol EvaGreen qPCR Mix Plus (Solis BioDyne) in 384-well optical plates and run in a 7900HT Fast Real-Time PCR System (Applied Biosystems). Primer pairs Q27_F/Q28_R and Q30_F/Q31_R were used for positive and negative genome quantifications, respectively. The amount of target RNA in the analyzed samples was estimated by absolute quantification using an external DNA standard curve [114].

### MALDI MS/MS and data analysis

Plant total protein extracts were separated by glycine-SDS-PAGE and stained with Coomassie blue. Gel bands of interest were excised manually, reduced, alkylated and in-gel digested. The tryptic-eluted peptides were subjected to MALDI-TOF/TOF analysis. Data were automatically acquired in an ABi 4800 MALDI TOF/TOF mass spectrometer (AB Sciex) and searched against NCBI non-redundant protein database, NCBInr_20121116. Mass tolerance for precursors was set to ±50 ppm and for MS/MS fragment ions to ±0.3 Da. The confidence interval for protein identification was set to ≥95% (*p*<0.05) and only peptides with an individual ion score above the identity threshold were considered correctly identified.

Manual peptide *de novo* sequencing was performed according Ma and Johnson [115]. Residues with near/isobaric masses were *bona fide* assigned according alignment consensus. Confidence of retrieved results was further tested with Peaks Studio software (BSI) [116].

### iTRAQ sample labeling, LC-MS/MS and data analysis

*N. benthamiana* NahG plants were agro-inoculated with two independent bacteria cultures per each viral cDNA clone (*n* = 2 biological replicates); upper non-inoculated leaves were collected from 8 plants per culture (12 dpi). Total protein extracts were prepared as described for western blot assays and further purified by methanol/chloroform precipitation. Protein pellets were resuspended in 6 M guanidine hydrochloride and 100 mM HEPES, pH 7.5, and concentration was determined by RC DC assay (BioRad). Equal amounts of protein for each condition were trypsin-digested and labeled with iTRAQ Reagent Multi-plex kit (AB Sciex). Tags 114 and 116 were used for P1Pro biological replicates and 115 and 117 for wild-type PPV biological replicates. Labeled peptide samples were combined and subjected to LC-MS/MS analysis (three technical replicates) using a nano liquid chromatography system (Eksigent Technologies) coupled to a Triple TOF 5600 mass spectrometer (AB Sciex). MS and MS/MS data were processed using Analyst TF 1.5.1 software (AB Sciex) and searched using the Mascot Server v. 2.3.02 (Matrix Science) against a customized database represented by *N. benthamiana* genome-predicted proteins (available at Sol Genomics Network [59]) plus their corresponding reversed entries. Peptide mass tolerance was set to ±20 ppm for precursors and 0.05 Da for fragment masses. The confidence interval for protein identification was set to ≥95% (*p*<0.05), and only peptides with an individual ion score above the identity threshold were maintained in quantification analysis. Proteins were considered differentially expressed if they had at least two quantified peptides and they were present in both P1Pro biological replicates with a false discovery rate <5%. LOG2 ratios relative to PPV biological replica #A (iTRAQ tag 115) were visualized by MultiExperiment Viewer [117]. Hierarchical clustering analysis with Pearson correlation distance metric was used to build the sample tree [118]. Sequences of reliably quantified *N. benthamiana* proteins were used as query in a WU-BLAST2 search against the TAIR10 protein dataset, to generate a list of homologous *A. thaliana* gene IDs. This was used as input in gene ontology term enrichment analysis using BinGO [119].

### Accession numbers

The following GenBank (http://www.ncbi.nlm.nih.gov) accessions were used in the viral sequence analysis: YMV (YP_022752.1); PVV (NP_734369.1); PVY (NP_734243.1); VVY (YP_001931974.1); CaYSV (YP_003208051.1); JGMV (NP_734408.1); MDMV (NP_734143.1); SCMV (NP_734133.1); SrMV (CAC84438.1); PVA (NP_734359.1); TEV (NP_734207.1); ScaMV (NP_734123.1); TuMV (BAC02772.1). Plant GenBank accessions considered: *N. tabacum* class II PR-2 (P23547.1); *N. tabacum* class I PR-2 (AAA63541.1); *S. tuberosum* class II PR-2 (CAE52322.1); *S. lycopersicum* class II PR-2 (NP_001234798.1). *N. benthamiana* accessions can be found at Sol Genomics Network (ftp://ftp.solgenomics.net/genomes/Nicotiana_benthamiana/annotation/).

## Supporting Information

Figure S1 **Alignment and protein disorder prediction of the six smallest known potyviral P1 sequences.** GenBank accession numbers are reported in Table S1, in Text S1; for PPV P1, only resides 164–308 were considered. Amino acid background is assigned according the ClustalX color scheme [120]. Residues aligning to PPV P1 minimal protease domain are shown in black letters and bright-colored background. The VELI motif is boxed; The protease conserved RG dipeptide is marked with black bar and the catalytic triad His, Asp and Ser is marked with stars. MD2 lines show protein disorder prediction of P1 sequence according MetadisorderMD2, where “+” are disordered and “−” ordered residues. DISOPRED2 prediction confidence is plotted: ScaMV, green line; SrMV, purple; SCMV, orange; JGMV, blue; MDMV, turquoise; CaYSV, magenta.
(TIF)

Figure S2 **Pseudo-reversions of P1 VE-189,190-AA mutations and infectivity of PPV ST2A viral clone.** *N. clevelandii* plants were agro-inoculated with wild-type PPV and viral clone VE (P1 VE-189,190-AA), clone S (P1 S259 replaced by alanine) and clone ST2A (P1 S259A+T2A peptide). (**A**) Anti-PPV HCPro (HC) western blot assay of upper non-inoculated leaves, collected at 22 dpi. For each clone, both samples shown in Figure 4C were analyzed together. Ponceau red-stained blot is shown as loading control. (**B**) RT-PCR analysis of plants challenged with VE mutant clone (22 dpi). A PPV cDNA fragment encompassing the mutated region (nt 1–1197 of the PPV genome) was amplified by RT-PCR from a pool of two plants and analyzed by DNA sequencing. Sanger sequencing results of the RT-PCR fragment and of the inoculated plasmid DNA are shown. The wild-type PPV sequence is shown as reference. Nucleotides that differ from the wild-type sequence are indicated in lower case. The nucleotide that leads to reversion of the amino acid codon is underlined. Encoded amino acids are indicated beneath each nucleotide sequence and between box-drawing characters. Mutated residues are in italic (alanine), reverted residue underlined. (**C**) GFP-fluorescence pictures of upper non-inoculated *N. clevelandii* leaves were taken in an epifluorescence microscope (22 dpi). Scale bar, 1 cm. (**D**) Anti-PPV CP (CP) western blot assay of upper non-inoculated leaves (22 dpi); each lane corresponds to a single plant sample. Ponceau red-stained blot is shown as loading control.
(TIF)

Figure S3 **HCPro silencing suppression activity is maintained in the absence of the P1 N-terminal region.** Transient RNA silencing assay was done by co-infiltrating leaf tissue with an *Agrobacterium* p35S:GFP culture and cultures of *Agrobacterium* pSN.5 P1-S (pS, producing P1 S259A+HCPro), pSN.5 PPV (pWT, producing wild-type P1+HCPro), or pSN.5 P1Pro (pP1Pro, producing P1 lacking amino acids 2−163+HCPro). (**A**) Picture of *N. benthamiana* agro-infiltrated leaf was taken in an epifluorescence microscope (6 dpa). (**B**) GFP fluorescent intensity (FI) from *N. benthamiana* infiltrated leaf patches was quantified in a fluorometer and plotted using average pWT value at 6 dpa equal to 100. Line graph shows mean ± SD (*n* = 16 samples/condition, from two independent *Agrobacterium* cultures); colors as in (A). For each time point, the mean GFP FI value (*n* = 16) from untreated leaves was used as blank. Analyzed time points (dpa) in the *x* axis. (**C**) Western blot detection of GFP protein in *N. clevelandii* samples (6 dpa); each lane represents a pool of samples from 2/3 infiltrated patches. N, non-infiltrated leaf sample. Ponceau red staining is shown as loading control. (**D**) Relative signal quantification for the immunoblot shown in (C) using average pWT value equal to 100. Histogram shows mean ± SD (*n* = 6 samples/condition, from two independent *Agrobacterium* cultures); ns, not significant by Student's *t*-test.
(TIF)

Figure S4 ***Nicotiana clevelandii*** **class II PR-2 peptide sequences assigned by MS/MS analysis.** (**A**) Identified sequence of MALDI-TOF peptides labeled in Figure 8C. Mass measurement accuracy of the retrieved peptides is indicated in parts per million (ppm). In *de novo* defined peptides, residues that differ from tobacco class II PR-2 isoform GI9 sequence (GenBank accession no. P23547.1) are in bold and underlined. Amino acids with near-/isobaric masses were *bona fide* assigned according to alignment consensus of the following panel. (**B**) Alignment of reference PR-2 sequences with *N. clevelandii* peptides (shown above the alignment). Residues that align with the peptides identified are boxed; background according the ClustalX color scheme [120]. In the rest of the alignment, only K/R and P are colored to highlight possible trypsin cleavage sites. Ntab_PR-2II, tobacco class II PR-2 isoform GI9 (GenBank P23547.1); Nben_PR-2II, *N. benthamiana* class II PR-2 predicted protein (Sol Genomics NbC25742659g0003.1); Stub_PR-2II, *Solanum tuberosum* class II PR-2 (GenBank CAE52322.1); Slyc_PR-2II, *S. lycopersicum* class II PR-2 (GenBank NP_001234798.1); Ntab_PR-2I, tobacco class I PR-2 clone gglb50 (GenBank AAA63541.1) as outlier. The last 22 residues of Ntab_PR-2I are hidden, as they do not align to the other accessions. In each alignment lane, positions of the first and last accession amino acids are indicated. In *de novo* defined peptides, residues that differ from the Ntab_PR-2II sequence are marked with a star.
(TIF)

Text S1 **This file contains: Supplemental Methods; Supplemental References; Table S1, Virus species and GenBank accession numbers for viral protein sequences used in the study; Table S2, List of the primers used in the study.**
(PDF)
