# Supplementary figures and images for: The Hypervariable Amino-Terminus of P1 Protease Modulates Potyviral Replication and Host Defense Responses
Source: PLoS Pathog. 2014 Mar 6;10(3):e1003985. doi: 10.1371/journal.ppat.1003985 (PMC3946448; doi:10.1371/journal.ppat.1003985)

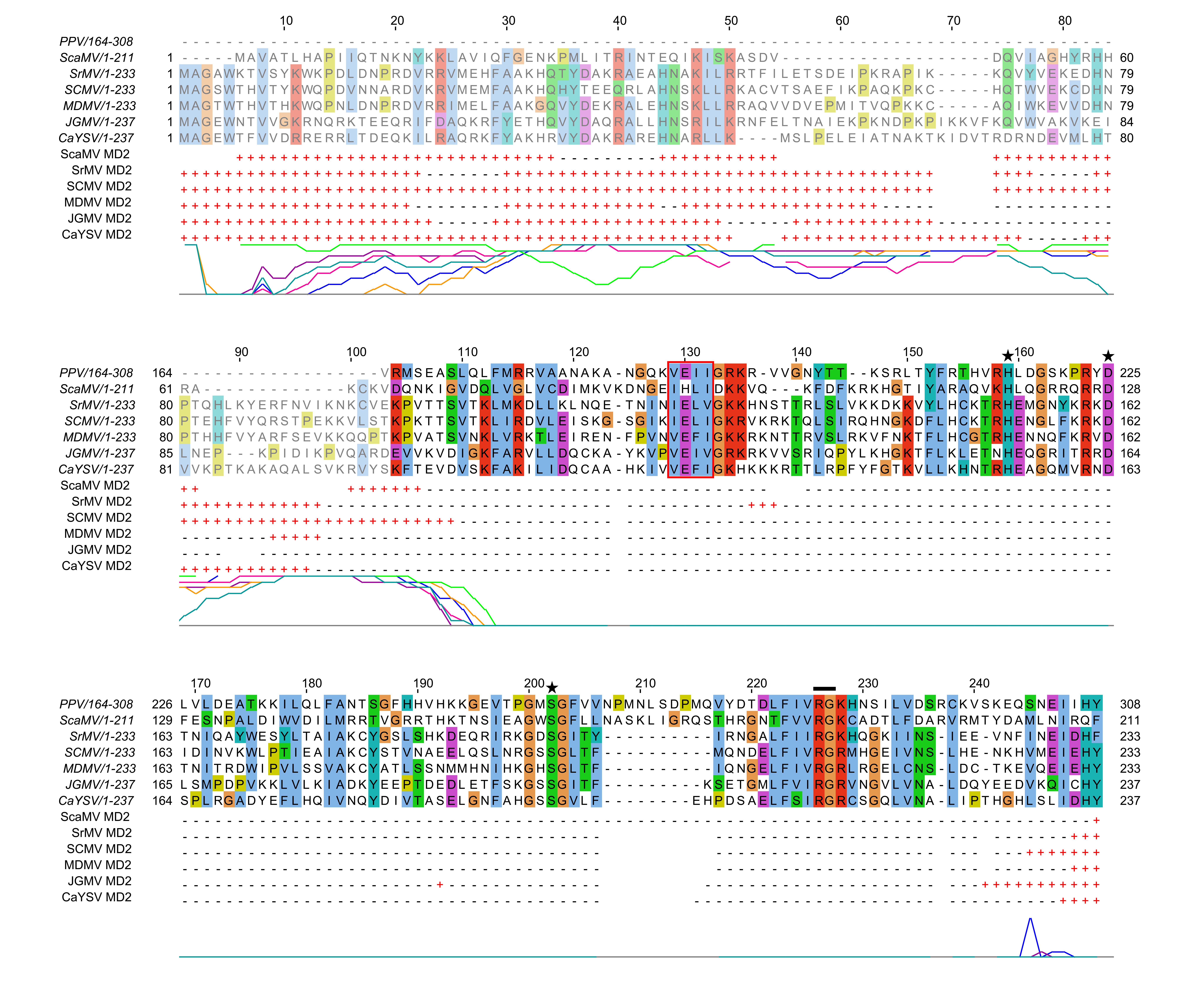

Supplement: Figure S1 — Alignment and protein disorder prediction of the six smallest known potyviral P1 sequences. GenBank accession numbers are reported in Table S1, in Text S1; for PPV P1, only resides 164–308 were considered. Amino acid background is assigned according the ClustalX color scheme [120]. Residues aligning to PPV P1 minimal protease domain are shown in black letters and bright-colored background. The VELI motif is boxed; The protease conserved RG dipeptide is marked with black bar and the catalytic triad His, Asp and Ser is marked with stars. MD2 lines show protein disorder prediction of P1 sequence according MetadisorderMD2, where “+” are disordered and “−” ordered residues. DISOPRED2 prediction confidence is plotted: ScaMV, green line; SrMV, purple; SCMV, orange; JGMV, blue; MDMV, turquoise; CaYSV, magenta. (TIF) [file ppat.1003985.s001.tif]

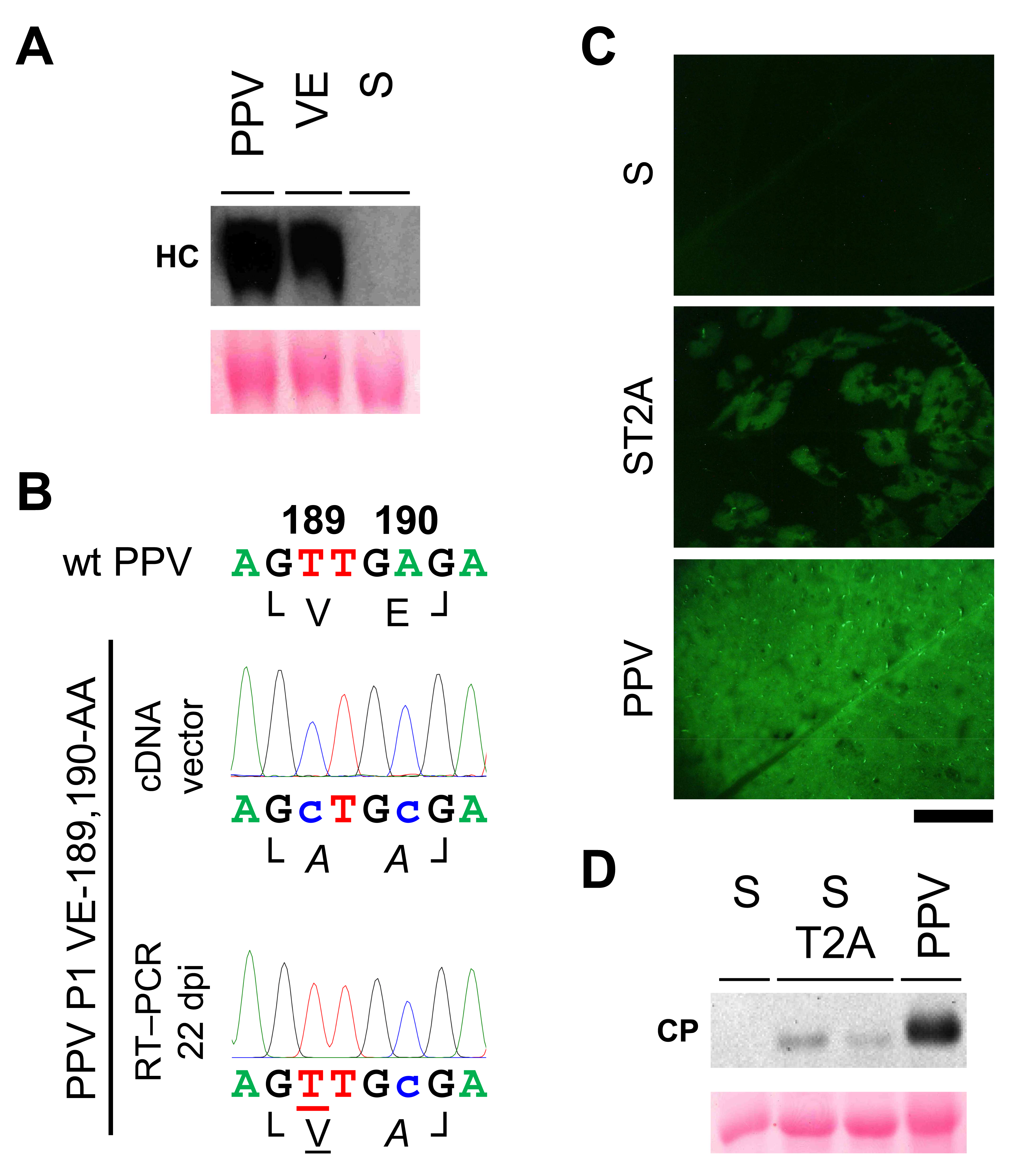

Supplement: Figure S2 — Pseudo-reversions of P1 VE-189,190-AA mutations and infectivity of PPV ST2A viral clone. N. clevelandii plants were agro-inoculated with wild-type PPV and viral clone VE (P1 VE-189,190-AA), clone S (P1 S259 replaced by alanine) and clone ST2A (P1 S259A+T2A peptide). (A) Anti-PPV HCPro (HC) western blot assay of upper non-inoculated leaves, collected at 22 dpi. For each clone, both samples shown in Figure 4C were analyzed together. Ponceau red-stained blot is shown as loading control. (B) RT-PCR analysis of plants challenged with VE mutant clone (22 dpi). A PPV cDNA fragment encompassing the mutated region (nt 1–1197 of the PPV genome) was amplified by RT-PCR from a pool of two plants and analyzed by DNA sequencing. Sanger sequencing results of the RT-PCR fragment and of the inoculated plasmid DNA are shown. The wild-type PPV sequence is shown as reference. Nucleotides that differ from the wild-type sequence are indicated in lower case. The nucleotide that leads to reversion of the amino acid codon is underlined. Encoded amino acids are indicated beneath each nucleotide sequence and between box-drawing characters. Mutated residues are in italic (alanine), reverted residue underlined. (C) GFP-fluorescence pictures of upper non-inoculated N. clevelandii leaves were taken in an epifluorescence microscope (22 dpi). Scale bar, 1 cm. (D) Anti-PPV CP (CP) western blot assay of upper non-inoculated leaves (22 dpi); each lane corresponds to a single plant sample. Ponceau red-stained blot is shown as loading control. (TIF) [file ppat.1003985.s002.tif]

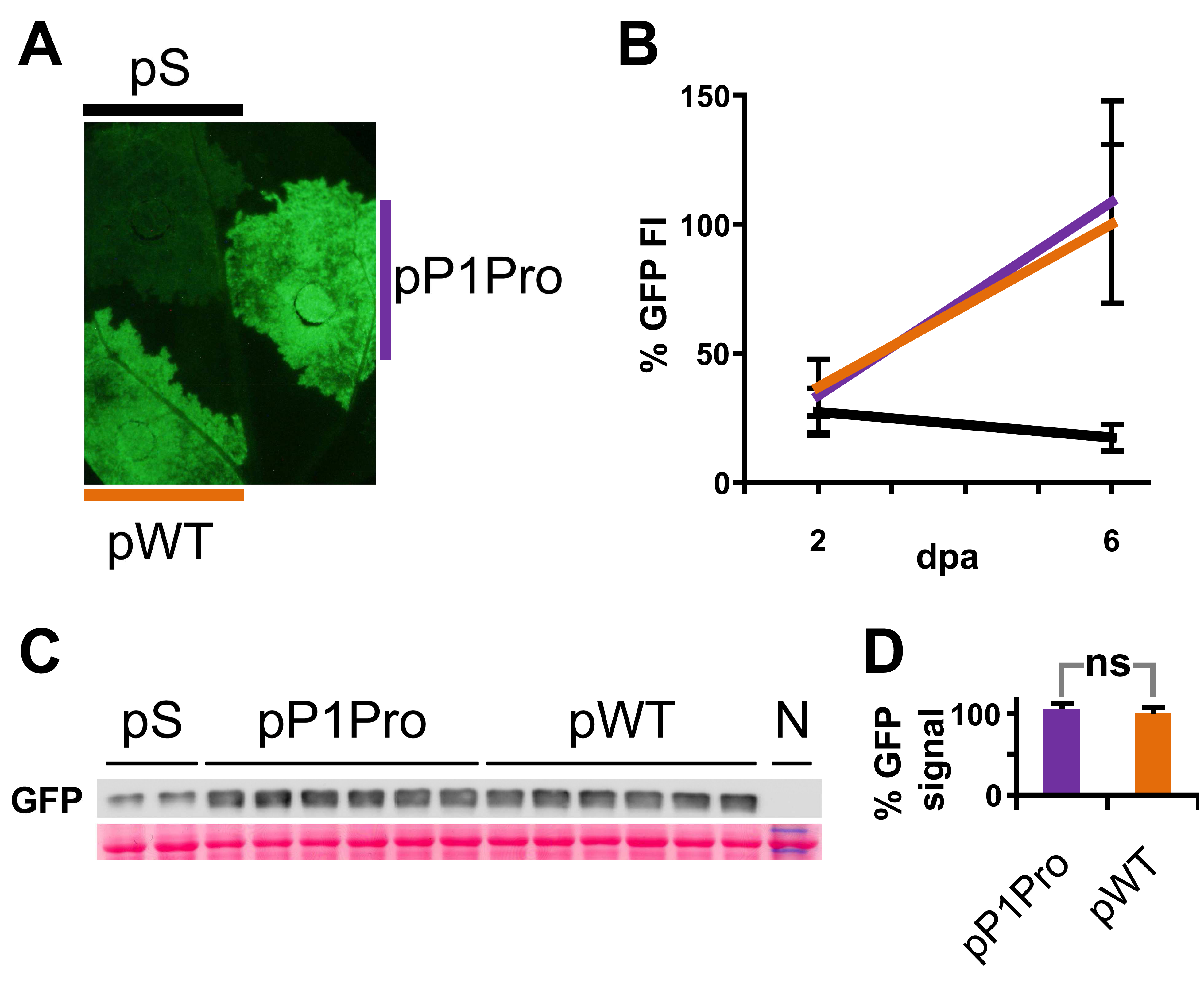

Supplement: Figure S3 — HCPro silencing suppression activity is maintained in the absence of the P1 N-terminal region. Transient RNA silencing assay was done by co-infiltrating leaf tissue with an Agrobacterium p35S:GFP culture and cultures of Agrobacterium pSN.5 P1-S (pS, producing P1 S259A+HCPro), pSN.5 PPV (pWT, producing wild-type P1+HCPro), or pSN.5 P1Pro (pP1Pro, producing P1 lacking amino acids 2−163+HCPro). (A) Picture of N. benthamiana agro-infiltrated leaf was taken in an epifluorescence microscope (6 dpa). (B) GFP fluorescent intensity (FI) from N. benthamiana infiltrated leaf patches was quantified in a fluorometer and plotted using average pWT value at 6 dpa equal to 100. Line graph shows mean ± SD (n = 16 samples/condition, from two independent Agrobacterium cultures); colors as in (A). For each time point, the mean GFP FI value (n = 16) from untreated leaves was used as blank. Analyzed time points (dpa) in the x axis. (C) Western blot detection of GFP protein in N. clevelandii samples (6 dpa); each lane represents a pool of samples from 2/3 infiltrated patches. N, non-infiltrated leaf sample. Ponceau red staining is shown as loading control. (D) Relative signal quantification for the immunoblot shown in (C) using average pWT value equal to 100. Histogram shows mean ± SD (n = 6 samples/condition, from two independent Agrobacterium cultures); ns, not significant by Student's t-test. (TIF) [file ppat.1003985.s003.tif]

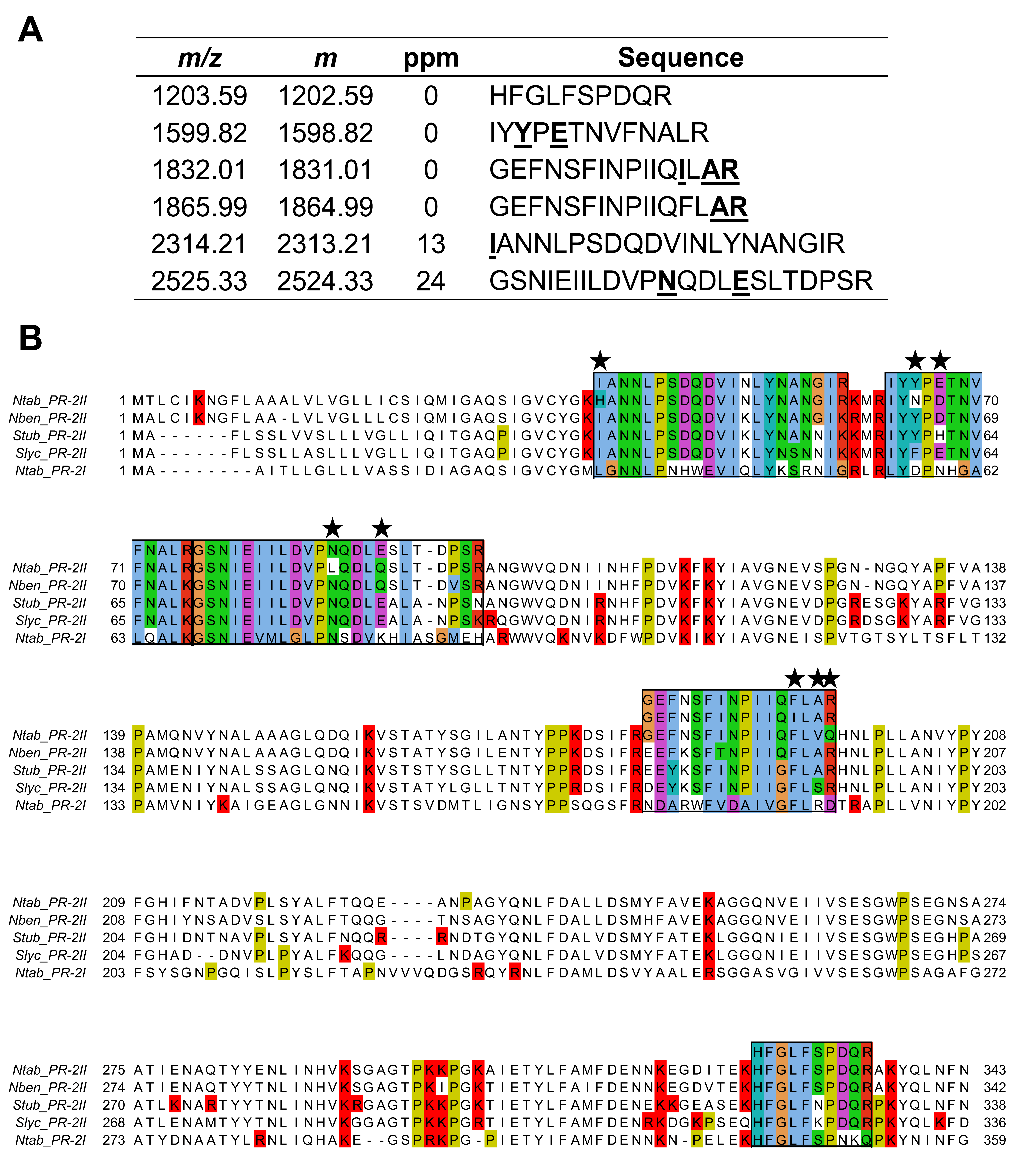

Supplement: Figure S4 — Nicotiana clevelandii class II PR-2 peptide sequences assigned by MS/MS analysis. (A) Identified sequence of MALDI-TOF peptides labeled in Figure 8C. Mass measurement accuracy of the retrieved peptides is indicated in parts per million (ppm). In de novo defined peptides, residues that differ from tobacco class II PR-2 isoform GI9 sequence (GenBank accession no. P23547.1) are in bold and underlined. Amino acids with near-/isobaric masses were bona fide assigned according to alignment consensus of the following panel. (B) Alignment of reference PR-2 sequences with N. clevelandii peptides (shown above the alignment). Residues that align with the peptides identified are boxed; background according the ClustalX color scheme [120]. In the rest of the alignment, only K/R and P are colored to highlight possible trypsin cleavage sites. Ntab_PR-2II, tobacco class II PR-2 isoform GI9 (GenBank P23547.1); Nben_PR-2II, N. benthamiana class II PR-2 predicted protein (Sol Genomics NbC25742659g0003.1); Stub_PR-2II, Solanum tuberosum class II PR-2 (GenBank CAE52322.1); Slyc_PR-2II, S. lycopersicum class II PR-2 (GenBank NP_001234798.1); Ntab_PR-2I, tobacco class I PR-2 clone gglb50 (GenBank AAA63541.1) as outlier. The last 22 residues of Ntab_PR-2I are hidden, as they do not align to the other accessions. In each alignment lane, positions of the first and last accession amino acids are indicated. In de novo defined peptides, residues that differ from the Ntab_PR-2II sequence are marked with a star. (TIF) [file ppat.1003985.s004.tif]
