# Supplementary material for: The Hypervariable Amino-Terminus of P1 Protease Modulates Potyviral Replication and Host Defense Responses
Source: PLoS Pathog. 2014 Mar 6;10(3):e1003985. doi: 10.1371/journal.ppat.1003985 (PMC3946448; doi:10.1371/journal.ppat.1003985)
Supplement: Text S1 — This file contains: Supplemental Methods; Supplemental References; Table S1, Virus species and GenBank accession numbers for viral protein sequences used in the study; Table S2, List of the primers used in the study. (PDF) [file ppat.1003985.s005.pdf]

Text S1 of Supporting Information

Supplemental Methods

DNA plasmids and constructs

Unless otherwise indicated, standard molecular cloning methods were used [1] and PCR reactions performed with Phusion High-Fidelity DNA Polymerase (Fermentas or New England BioLabs). The accuracy of all newly built plasmids was verified by restriction digestion analysis and DNA sequencing of PCR generated inserts and Gibson assembly sites. Primers were synthesized by Sigma-Aldrich; sequences are listed in Table S2.

pSN-ccdB binary vector

For cloning purposes, an intermediate binary vector pSN-ccdB was generated as follows. HCPro fragment was PCR-amplified from pIC-PPVnkGFP [2] using 1848_F/1849_R primers, column-purified, digested with BglII/XhoI/DpnI, and then gel-purified. A cassette containing the *ccd*B toxin and chloramphenicol resistance gene was amplified from pDONR207 (Life Technologies) using primers 1852_F/1853_R, column-purified, digested with BglII/XbaI/DpnI, and gel-purified. A fragment containing the replication origin and gentamicin resistance gene was amplified from pDONR207 using primers 1850_F/1851_R, column-purified, digested with XmaI/XhoI/DpnI, and gel purified. The product of ligation of the three fragments was transformed to One Shot *ccd*B Survival (Life Technologies) and plasmid pSLIC Δ35S obtained. A fragment containing the CaMV 2x35S promoter was amplified from pIC-PPVnkGFP using primers 1842_F/1843_R and AmpliTaq DNA Polymerase (Life Technologies), gel-purified, mixed with pSLIC Δ35S and overlap extension PCR cloning [3] performed. The derived plasmid pSLIC 2x35S

was used as template for two independent PCR reactions, one with primers 1927_F/1928_R and another one with primers 1929_F/1930_R. Both reactions were gel-purified, mixed in equal amounts, denatured for 5 min at 98°C, and cooled slowly to room temperature as reported [4]. The insert was mixed in 1x T4 DNA ligase buffer with pBINPPV-NK-GFP [5], previously digested with ScaI/SnaBI and treated with T4 DNA polymerase. The mixture was heated for 15 min at 75°C, cooled slowly to room temperature, T4 DNA ligase added and transformed to One Shot *ccd*B Survival to obtain pSN-ccdB. The plasmid harbors a full-length cDNA copy of the PPV genome, except the 5'UTR-P1 region that was replaced by the *ccd*B gene flanked by two SfaAI restriction sites. As the parental vector, the sGFP(S65T) [6] sequence is present between NIb and CP.

Viral clone vectors

The pONE plasmid containing one copy of CaMV 35S and the PPV 5'UTR-P1 sequence, was generated by ligation of a PCR performed with primers 2018_F/1849_R on pIC-PPVnkGFP, followed by digestion with XmaI/XhoI/DpnI and a second PCR performed with primers 1850_F/1851_R on pDONR207, followed by digestion with XmaI/XhoI/DpnI.

pSN-PPV. The fragment obtained by PCR with primers 1986_F/1910_R on pONE was gel-purified, mixed in 2:1 molar ratio with pSN-ccdB previously linearized by SfaAI and purified by organic extraction/EtOH precipitation. The DNA mixture was used in a Gibson one-step isothermal DNA assembly reaction [7] containing (final concentrations) 100 mM Tris-HCl pH 7.5, 10 mM $MgCl_2$, 0.2 mM dNTP mix, 10.0 mM DTT, 5.0% w/v PEG-8000, 1.0 mM NAD+, 0.004 U/µL T5 exonuclease, 0.025 U/µL Phusion High-Fidelity DNA Polymerase, 4.0 U/µL Taq DNA ligase. Similarly, PCR fragments containing deletions or point mutations in the 5'UTR-P1 region were

generated by gene splicing *via* the overlap extension method [8], as detailed below. Amplicons obtained were inserted in SfaAI-digested pSN-ccdB by Gibson assembly, as described.

pSN-PPV ΔP1, clone with no P1 sequence. pONE was used as template for two independent PCRs with primers 1986_F/2063_R (to obtain the 5'ΔP1 fragment) and 2064_F/1930_R (to obtain the 3'ΔP1 fragment); the purified fragments were joined by fusion PCR with 1986_F/1910_R.

pSN-PPV P1-FG, with P1 FG-6,7-AA amino acid substitutions. pONE was used as template for two independent PCRs with primers 1986_F/2073_R and 2072_F/1910_R.

pSN-PPV P1-WE, with P1 W63A E67A substitutions. pONE was used as template for two independent PCRs with primers 1986_F/2075_R and 2074_F/1910_R.

pSN-PPV P1-VE, with P1 VE-189,190-AA substitutions. pONE was used as template for two independent PCRs with primers 1986_F/2077_R and 2076_F/1910_R.

pSN-PPV P1-S, with P1 S259A substitution. pONE was used as template for two independent PCRs with primers 1986_F/2079_R (to obtain the 5'P1-S fragment) and 2078_F/1930_R; the purified fragments were joined by fusion PCR with 1986_F/1910_R.

pSN-PPV P1-HY, with P1 HY-307,308-AA substitutions. pONE was used as template for two independent PCRs with primers 1986_F/2081_R and 2080_F/1930_R; the purified fragments were joined by fusion PCR with 1986_F/1910_R.

pSN-PPV P1-ST2A, with P1 S259A substitution and *Thosea asigna* virus 2A [9] "self-cleaving" peptide RAEGRGSLLTCGDVEENPG↓P (T2A) inserted between P1 and HCPro. pONE was used as template for two independent PCRs with primers 1986_F/2083_R and 2082_F/1930_R; the purified fragments were used as template in a fusion PCR with 2078_F/1910_R. The purified fragment was joined to the 5'P1-S fragment by fusion PCR with 1986_F/1910_R.

pSN-PPV ∆P1-AAG, clone with no P1 sequence and PPV 5'UTR t-37-a nucleotide substitution to mutate the in-phase ATG upstream of the polyprotein starting codon. pONE was used as template for a PCR with primers 1986_F/2067_R, to obtain the 5'AAG fragment. The 5'∆P1 and 3'∆P1 fragments were used as template in a fusion PCR with 2066_F/1930_R, to obtain the 3'AAG fragment. Purified 5'AAG and 3'AAG fragments were joined by fusion PCR with 1986_F/1910_R.

pSN-PPV P1Pro[V164], clone in which the P1 2-163 residues were deleted. pONE was used as template for a PCR with primers 1986_F/2063_R. A second PCR fragment was amplified from pIC-PPVnkGFP using 2229_F/2173_R. The purified fragments were joined by fusion PCR with 1986_F/1910_R.

In vitro transcription constructs

T7_P1 wt. pSN-PPV ∆P1-AAG was used as template for a PCR with primers 1985_F/2063_R, to obtain the PPV 5'UTR fragment. pIC-PPVnkGFP was used as template for a PCR with primers 1630_F/1632_R, to obtain the PPV 3'UTR fragment. A construct-specific fragment containing the full-length P1 and the sequence of the first 97 amino acids of HCPro (HC-97) followed by a stop codon was amplified from pIC-PPVnkGFP with 2174_F/2173_R. The three PCR products were DpnI-treated, gel-purified and joined in a PCR with 2172_F/1632_R. In the final fusion product, the

P1|HC-97 coding sequence is driven by the T7 RNA polymerase promoter and flanked by both PPV 5'UTR and 3'UTR. Similarly, transcription templates with deletions or point mutations in the P1 region were generated by performing the final fusion PCR with the construct-specific fragments obtained as follows:

T7_P1-FG. PCR fragment amplified from pSN-PPV P1-FG using 2174_F/2173_R.

T7_P1-WE. PCR fragment amplified from pSN-PPV P1-WE using 2174_F/2173_R.

T7_P1-VE. PCR fragment amplified from pSN-PPV P1-VE using 2174_F/2173_R.

T7_P1-S. PCR fragment amplified from pSN-PPV P1-S using 2174_F/2173_R.

T7_P1-ST2A. PCR fragment amplified from pSN-PPV P1-ST2A using 2174_F/2173_R.

T7_P1-HY. PCR fragment amplified from pSN-PPV P1-HY using 2174_F/2173_R.

T7_P1Pro-T162. PCR fragment amplified from pIC-PPVnkGFP using 2175_F/2173_R.

T7_P1Pro-T162 S. PCR fragment amplified from pSN-PPV P1-S using 2175_F/2173_R.

T7_P1Pro-K163. PCR fragment amplified from pIC-PPVnkGFP using 2230_F/2173_R.

T7_P1Pro-V164. PCR fragment amplified from pIC-PPVnkGFP using 2229_F/2173_R.

T7_P1Pro-R165. PCR fragment amplified from pIC-PPVnkGFP using 2228_F/2173_R.

T7_P1Pro-M166. PCR fragment amplified from pIC-PPVnkGFP using 2227_F/2173_R.

T7_P1Pro-S167. PCR fragment amplified from pIC-PPVnkGFP using 2226_F/2173_R.

T7_P1Pro-E168. PCR fragment amplified from pIC-PPVnkGFP using 2225_F/2173_R.

T7_P1Pro-A169. PCR fragment amplified from pIC-PPVnkGFP using 2224_F/2173_R.

T7_P1Pro-S170. PCR fragment amplified from pIC-PPVnkGFP using 2176_F/2173_R.

T7_P1Pro-V178. PCR fragment amplified from pIC-PPVnkGFP using 2177_F/2173_R.

The specificity of PCR-generated DNA constructs was confirmed by gel electrophoresis, and constructs were used directly in *in vitro* transcription reactions.

Transient expression vectors

A first PCR fragment was amplified from pSITEII-2C1 [10] using 2254_F/2255_R; a second PCR fragment was amplified from pSITEII-2C1 using 2256_F/2257_R; a third PCR fragment was amplified from pIC-PPVnkGFP using 2258_F/2259_R. The purified amplicons were joined by fusion PCR with 2254_F/2259_R, to obtain the EGFP fragment. pIC-PPVnkGFP was used as template for two independent PCRs with primers 2250_F/2251_R and 2252_F/2253_R, and the purified amplicons were joined by fusion PCR with 2250_F/2253_R to obtain the PPV 3'UTR fragment. Both EGFP and PPV 3'UTR fragments were mixed in equal molar amounts with HindIII/SpeI-digested pSITEII-2C1 and subjected to Gibson assembly to obtain

pSN.5-GFP. pSN.5-GFP was digested with BstBI/SpeI and combined with BstBI/SpeI-digested pSN-ccdB to obtain pSN2-ccdB.

pSN2-ccdB was digested with XbaI/BstBI and combined with SpeI/BstBI-digested pSN-PPV to obtain pSN.5 PPV, with SpeI/BstBI-digested pSN-PPV P1-S to obtain pSN.5 P1-S, with SpeI/BstBI-digested pSN-PPV P1-ST2A to obtain pSN.5 P1-ST2A, and with SpeI/BstBI-digested pSN-PPV P1Pro[V164] to obtain pSN.5 P1Pro.

Agrobacterium tumefaciens C58C1 strain carrying p35S:GFP [11] plus pCH32 [12] was kindly provided by Prof. D. Baulcombe (University of Cambridge, UK).

Supplemental References

1. Sambrook J, Russel DW (2001) Molecular cloning: a laboratory manual (3rd edition). Cold Spring Harbor Laboratory Press.
2. Fernández-Fernández MR, Mouriño M, Rivera J, Rodríguez F, Plana-Durán J, et al. (2001) Protection of rabbits against rabbit hemorrhagic disease virus by immunization with the VP60 protein expressed in plants with a potyvirus-based vector. Virology 280: 283–291. doi:10.1006/viro.2000.0762.
3. Bryksin A, Matsumura I (2010) Overlap extension PCR cloning: a simple and reliable way to create recombinant plasmids. Biotechniques 48: 463–465. doi:10.2144/000113418.
4. Li MZ, Elledge SJ (2007) Harnessing homologous recombination in vitro to generate recombinant DNA via SLIC. Nat Methods 4: 251–256. doi:10.1038/nmeth1010.
5. Lucini C (2004) Expresión de proteínas heterólogas en plantas por medio del virus de la sharka (PPV) Madrid: Universidad Politécnica de Madrid.
6. Chiu W, Niwa Y, Zeng W, Hirano T, Kobayashi H, et al. (1996) Engineered GFP as a vital reporter in plants. Curr Biol 6: 325–330. doi:10.1016/S0960-9822(02)00483-9.
7. Gibson DG, Young L, Chuang R-Y, Venter JC, Hutchison III CA, et al. (2009) Enzymatic assembly of DNA molecules up to several hundred kilobases. Nat Methods 6: 343–345. doi:10.1038/nmeth.1318.
8. Horton RM, Hunt HD, Ho SN, Pullen JK, Pease LR (1989) Engineering hybrid genes without the use of restriction enzymes: gene splicing by overlap extension. Gene 77: 61–68. doi:10.1016/0378-1119(89)90359-4.
9. Donnelly MLL, Hughes LE, Luke G, Mendoza H, ten Dam E, et al. (2001) The “cleavage” activities of foot-and-mouth disease virus 2A site-directed mutants and naturally occurring “2A-like” sequences. J Gen Virol 82: 1027 –1041.
10. Martin K, Kopperud K, Chakrabarty R, Banerjee R, Brooks R, et al. (2009) Transient expression in *Nicotiana benthamiana* fluorescent marker lines provides enhanced definition of protein localization, movement and interactions *in planta*. Plant J 59: 150–162. doi:10.1111/j.1365-313X.2009.03850.x.
11. Haseloff J, Siemering KR, Prasher DC, Hodge S (1997) Removal of a cryptic intron and subcellular localization of green fluorescent protein are required to mark transgenic *Arabidopsis* plants brightly. Proc Natl Acad Sci USA 94: 2122–2127.
12. Hamilton CM, Frary A, Lewis C, Tanksley SD (1996) Stable transfer of intact high molecular weight DNA into plant chromosomes. Proc Natl Acad Sci USA 93: 9975–9979.
13. Gibbs A, Ohshima K (2010) Potyviruses and the digital revolution. Annu Rev Phytopathol 48: 205–223. doi:10.1146/annurev-phyto-073009-114404.

Table S1. Virus species and GenBank accession numbers for viral protein sequences used in the study.

Acronym	Species	GenBank accession	P1 size aa	Species group[1]
PPV[2]	*Plum pox virus*		308	n.a.
YMV	*Yam mosaic virus*	YP_022752.1	297	n.a.
PVV	*Potato virus V*	NP_734369.1	289	PVY group
PVY	*Potato virus Y*	NP_734243.1	284	PVY group
VVY	*Verbena virus Y*	YP_001931974.1	285	PVY group
CaYSV	*Canna yellow streak virus*	YP_003208051.1	237	SCMV group
JGMV	*Johnsongrass mosaic virus*	NP_734408.1	237	SCMV group
MDMV	*Maize dwarf mosaic virus*	NP_734143.1	233	SCMV group
SCMV	*Sugarcane mosaic virus*	NP_734133.1	233	SCMV group
SrMV	*Sorghum mosaic virus*	CAC84438.1	233	SCMV group
PVA	*Potato virus A*	NP_734359.1	298	TEV group
TEV	*Tobacco etch virus*	NP_734207.1	304	TEV group
ScaMV	*Scallion mosaic virus*	NP_734123.1	211	TuMV group
TuMV	*Turnip mosaic virus*	BAC02772.1	362	TuMV group

[1] Species group as previously defined [13]; n.a., not applicable

[2] PPV P1 cDNA sequence from pIC-PPVnkGFP [2] was used

Table S2. List of the primers used in the study.

Primer	Sequence (5'-->3')	Application[1]
1595_F	GAAAACATAAGAACTCAACACAACA	R
1597_R	CAAGTCCTGCGACATAACCAC	R
1630_F	CAGACGAAGGCAGCAGCATTG	T
1632_R	GTTTTTTTTTTTTTTTTTGTCTCTTGC	T
1842_F	GGCCTTTTGCTCACATGTTCTTTCC	V
1843_R	CTGACGGATGGCCTTTTTGCGTTTCTAGAGCGATCGCAATGATGGCATTTGTAGGAG	V
1848_F	ATTCATTTATGCAGATCTGCGATCGCGAAACTGATCATCAGTGC	V
1849_R	TATTTCAGACTCGAGTGGTGTACCGTCTTCGTG	V
1850_F	TCTGTCGTCACTCGAGGCGATTTCTCACTTG	V
1851_R	TCCACAGAAACCCGGGATAACGCAGGAAAGAAC	V
1852_F	CCGGAAGAGTCTCTAGAAACGCAAAAAGGC	V
1853_R	CGACGCACTTAGATCTGAATAAATACCTGTGACG	V
1910_R	TGCTGCGACATAACCACACTC	V
1927_F	GCATCAAGAACACAGAGAAAGATATATTTCTCAAGATCAGAAGTACTAGTCCAGTACGCACGATTCAAG	V
1928_R	GTAGTGCTGCCAGATTTGTTGACATAACAAGG	V
1929_F	ACTAGTCCAGTACGCACGATTCAAGGCT	V
1930_R	GAGACCTCAAAATGAATGCACTCTTCACC	V
1985_F	AGAGGATTGACGTGATAACATGG	T
1986_F	GCCCAGCTATCTGTCACTTTATTG	V
2018_F	CGTATTAAAAGCCCGGGGATTGATGTGATAACATGG	V
2063_R	CATCTTGACTTGCAGTAAATTTGGTAG	V, T
2064_F	CCTTAATTTCTCTACCAAATTTACTGCAAGTCAAGATGTCAGACCCAGGCAAACAAT	V
2066_F	CTCAACACAACATACAAAATTTTAAGCAATCAAATCAATC	V
2067_R	GATTGATTTGATTGCTTAAAATTTTGTATGTTGTGTTGAG	V
2072_F	ATGTCAACCATTGTAGCTGCCTCATTCACTTGCCACCTCG	V
2073_R	CGAGGTGGCAAGTGAATGAGGCAGCTACAATGGTTGAC	V
2074_F	GCAACAGCTAGTGCTGCGAAAGGGCTGGCAGAGAAGTT	V
2075_R	GCAACTTCTCTGCCAGCCCTTTCGCAGCACTAGC	V
2076_F	GTCAAAAAGCTGCGATCATAGGGCGTAAG	V
2077_R	CCGACTACACGCTTACGCCCTATGATCGCAGCTTTTTGAC	V
2078_F	CAAGAAAGGGGAGGTAACACCAGGAATGGCCGGATTTGTG	V
2079_R	CCACAAATCCGGCCATTCCTGGTGTTAC	V
2080_F	AGAACAGAGCAATGAGATAATCGCCGCATCTGACCCAGGCAAAC	V

2081_R	CCTGGGTCAGATGCGGCGATTATCTCA	V
2082_F	GGGAGAGCAGAAGGGAGAGGAAGCTTGCTAACCTGTGGAGACGTTGAGGAAAATCCAGGGCCATCTG ACCCAGGCAAACAAT	V
2083_R	CCACAGGTTAGCAAGCTTCCTCTCCCTTCTGCTCTCCCGCCTCCGCCTCCAGGATCTGAGTAGTGGATT ATCTCATTGCTCTG	V
2172_F	CTATATAAGGAATAATACGACTCACTATAGGGAAAATATAAAAACTCAAC	T
2173_R	GAACATTTCTCAATGCTGCTGCCTTCGTCTGGCTAAGCGAGAACATGTGAAAATTG	V, T
2174_F	CCTTAATTTCTCTACCAAATTTACTGC	T
2175_F	CCTTAATTTCTCTACCAAATTTACTGCAAGTCAAGATGACTAAGGTTAGGATGTCCGAG	T
2176_F	CCTTAATTTCTCTACCAAATTTACTGCAAGTCAAGATGTCACTACAACTTTTTATGAGGAG	T
2177_F	CCTTAATTTCTCTACCAAATTTACTGCAAGTCAAGATGGTTGCTGCAAACGCCAAG	T
2224_F	CTACCAAATTTACTGCAAGTCAAGATGGCATCACTACAACTTTTTATGAG	T
2225_F	TACCAAATTTACTGCAAGTCAAGATGGAGGCATCACTACAACTTTTTATG	T
2226_F	TCTACCAAATTTACTGCAAGTCAAGATGTCAGAGGCATCACTACAACTTT	T
2227_F	CTCTACCAAATTTACTGCAAGTCAAGATGATGTCCGAGGCATCACTACAA	T
2228_F	TTCTCTACCAAATTTACTGCAAGTCAAGATGAGAATGTCCGAGGCATCAC	T
2229_F	TCTCTACCAAATTTACTGCAAGTCAAGATGGTTAGAATGTCCGAGGCATC	V, T
2230_F	TTCTCTACCAAATTTACTGCAAGTCAAGATGAAGGTTAGGATGTCCGAGG	T
2250_F	CCGGTACCACTAGTCGCACGGAGAACATTAGGAG	A
2251_R	CTTGTGTTTCCCCATCCATCATCACTATCCAACCAGGTATGTTTTCAT	A
2252_F	CTGGTTGGATAGTGATGATGGATGGGGAAA	A
2253_R	GCGGGAAACGACAATCTGATCCAAGCTCAAGCTAGAGATCGAGTAACATAGATGACACC	A
2254_F	TCCGCTCCTAATGTTCTCCGTGCGACTAGTGGTACCGGGCTGAAAGCGAC	A
2255_R	GTTTTGTATATGTTGTGTTGAGATTGGTAGATAGCAAATATAATGGT	A
2256_F	ATCTACCAATCTCAACACAACATATACAAAACAAACG	A
2257_R	AGGTCAGGGTGGTCACGAGGGT	A
2258_F	ACCCTCGTGACCACCCTGACCTACGGCGTGCAGTG	A
2259_R	CGTCTCGCATATCTCATTAAAGCAGGACTCTAGGGATCACTTGTAAAGCTCGTCCATGCCGTGAGT	A
Q26_R	TTTCGTCTTCTACTTGCTAACGGACTCTTGCTCCAATTCCTACAGATAAC	Q
Q27_F	ATCCTACACGATGGCAGTGAAAAC	Q
Q28_R	TCTTCTACTTGCTAACGGACTCTTG	Q
Q29_F	GCAACACCTCTACAATGTGATAACCGACTGATGCAGGAACTGGAGCAAGC	Q
Q30_F	CTCTACAATGTGATAACCGACTGATG	Q
Q31_R	CGCCGTCTTTTGGGATGAA	Q

[1] Application as follows: A, transient agro-infiltration assay plasmid preparation; Q, RT-qPCR; R, RT-PCR; T, *in vitro* translation construct; V, PPV cDNA clone engineering.
